# Conditions for Barrel and Clam-Shell Liquid Drops to Move on Bio-inspired Conical Wires

**DOI:** 10.1038/s41598-017-10036-3

**Published:** 2017-08-29

**Authors:** Cheng Luo, Xiang Wang

**Affiliations:** Department of Mechanical and Aerospace Engineering, University of Texas at Arlington 500 West First Street, Woolf Hall 226, Arlington, TX 76019 United States of America

## Abstract

It has been reported that, in a foggy environment, water drops with either barrel or clam-shell shapes are capable of self-running on conical wire-like structures, such as cactus spines, spider silk, and water striders’ legs. On the other hand, the corresponding moving mechanisms are still not quite understood. For instance, it is unclear under what conditions clam-shell drops would move from the tip towards the root on a conical wire. In this work, based on the balance of forces, we derive conditions for a drop to self-transport towards or away from the root. We find that, although barrel and clam-shell drops have different shapes, these conditions are applicable to both of them, which thus provide good guidelines for developing artificial fog collectors. Furthermore, based on the derived conditions, we interpret drop movements on both hydrophilic and hydrophobic wires, with the support of experimental results on cactus spines. Finally, our results indicate that not all the cacti are able to harvest water from fog.

## Introduction

**Figure 1:**
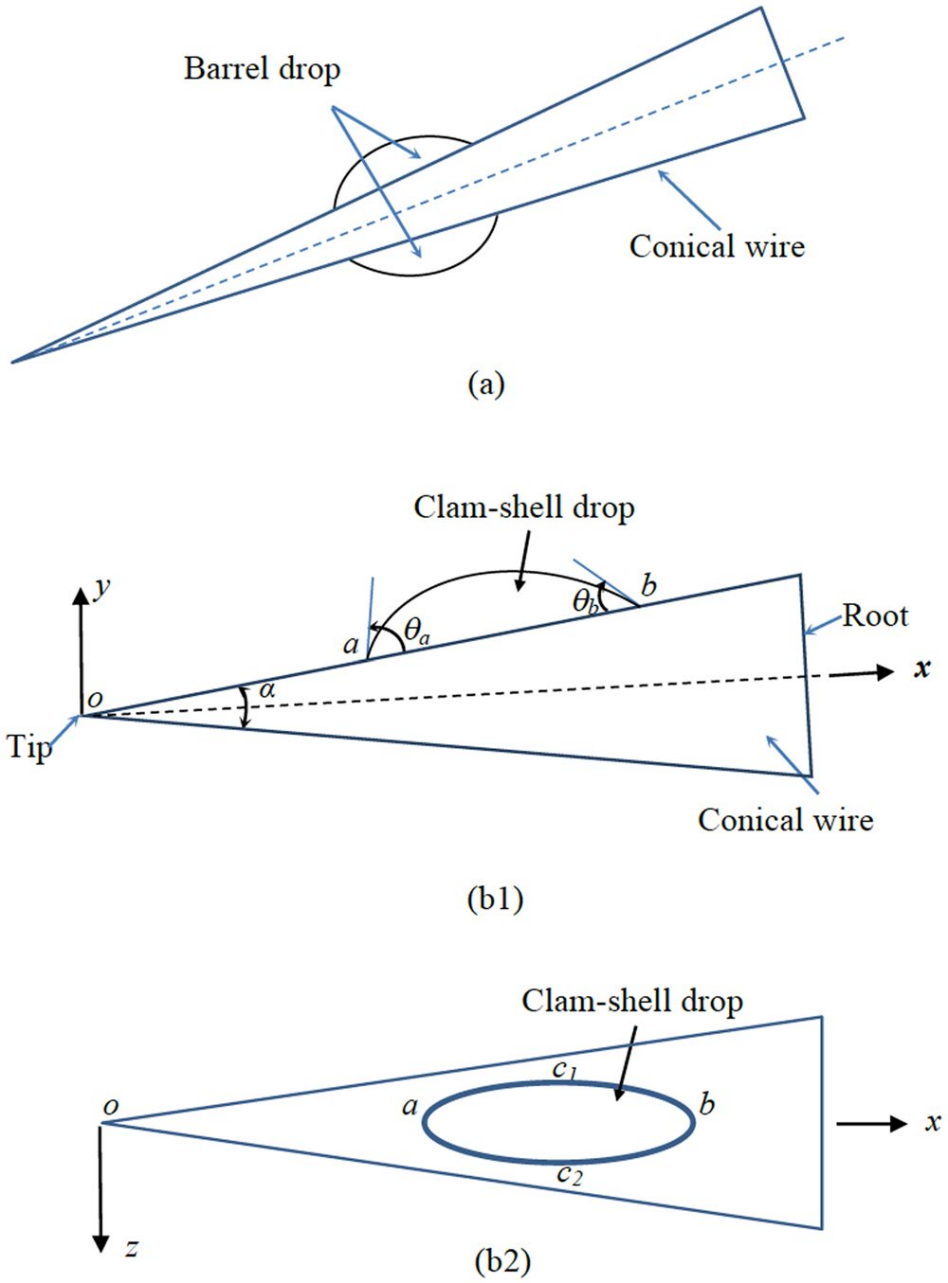
(**a**) A barrel drop on a conical wire. (**b1**) Cross-sectional and (**b2**) top views of a clam-shell drop at rest on a conical wire (schematics).

Water drops are capable of transporting on conical wire-like structures, such as cactus spines^1^, spider silks^2^ and water striders’ legs^3^, from narrow (i.e., tips) to wide ends (i.e., roots) of the structures. On a conical wire, liquid drops may exhibit two basic shapes: barrel and clam-shell (Fig. 1). The barrel drop is axisymmetric with respect to the central axis of the wire, while the clam-shell type has a more complicated profile, which is asymmetric. An inequality, which involves the drop size, contact angle, and wire radius, has been previously derived in ref 4 to identify whether a drop has a barrel or clam-shell shape. According to this inequality, a large drop may have a barrel shape on a thin wire with a wetting surface, while a small drop may exhibit a clam-shell profile on a thick wire with a non-wetting surface.

Through numerical calculation, Hanumanthu and Stebe determined equilibrium shapes and positions of barrel drops on conical wires^5^. Carrol considered a barrel drop on a thin cylinder, which had identical cross-sections^6^. He derived an analytical expression for its drop profile. To obtain the gradient of Laplace pressure, Lorenceau and Quéré further extended Carrol’s solution to the case of a thin conical wire^7^. With the aid of the derived pressure gradient, they explored driving mechanism of a barrel drop on the thin conical wire, when apparent contact angle was 0°^7^. Using a similar line of reasoning, we considered the case that the apparent contact angle was non-zero^8^. We found that these drops might move from the tip to root only on a lyophilic conical wire. However, it is unclear whether the same would apply to clam-shell drops.

In fog tests, the clam-shell drops are more often seen than the barrel type^1–3, 9^, and tiny drops that initially emerge on a conical wire usually have a clam-shell shape. On the other hand, little theoretical or numerical work has been done regarding moving conditions of clam-shell drops, except that Tan *et al*. have recently simulated their movements on conical wires in special cases^10^. This motivates us to further investigate the clam-shell drops.

In our previous consideration of a barrel drop, the difference in liquid pressure was used to derive conditions for the drop to move on a conical wire with a small conical angle^8^. Liquid pressure is related to two principal curvatures of the drop profile. In the case of barrel drops, due to their axisymmetric shapes, it is relatively easy to obtain these principal curvatures even without knowing analytical expression of their profiles^4, 6, 8, 11^. Nevertheless, clam-shell drops are asymmetric. This makes it difficult to find the principal curvatures directly according to the geometric relations. An alternative method is to make use of the analytical expression of the drop profile, which may be obtained by solving the associated Laplace equation. The second derivative of the corresponding analytical relation may give the expression of the principal curvatures. However, in contrast to the problem of a barrel drop, no analytical solution to Laplace equation is reported for the clam-shell drop except for numerical ones^12, 13^. Thus, it may not be a good option to derive the moving conditions for a clam-shell drop on the basis of the pressure difference. Consequently, a different approach is adopted here. This approach is based on the balance of forces on a drop, which avoids the need of finding an analytical expression for the drop profile.

### Balance of forces on a drop located on a conical wire

Consider a clam-shell drop on a conical wire (Fig. 1b). The wire surface is not limited to be smooth. As in the case of cactus spines^1, 14, 15^, it may be rough and include microgrooves and microbarbs. Let *o* denote the edge of the wire tip. Set *a*, *b*, *c*_1_ and *c*_2_ to be triple-phase (air/solid/liquid) contact points at the four ends of the interface between the drop and wire surface. Set up an *xyz* rectangular coordinate system. The origin is located at *o*. *x* axis is along the central axis of the wire. *y* axis is perpendicular to *x* axis, and lies in the same plane as *a* and *b*, and *z* axis is perpendicular to both *x* and *y* axes. Accordingly, the drop profile is symmetric with respect to *x*-*y* plane. Use *α* to stand for conical angle of the wire. Set *θ*_*a*_ and *θ*_*b*_ to be equilibrium contact angles at *c*_1_ *ac*_2_ and *c*_2_ *bc*_1_, respectively^16, 17^. When the wire surface is smooth, *θ*_*a*_ and *θ*_*b*_ are intrinsic contact angles. Otherwise, they are apparent contact angles. Let *θ*_*adv*_ and *θ*_*rec*_ respectively, stand for advancing and receding contact angles. Both *θ*_*a*_ and *θ*_*b*_ vary between *θ*_*rec*_ and *θ*_*adv*_. $({\theta }_{adv}-{\theta }_{rec})$ is so-called contact angle hysteresis. A triple-phase contact line is pinned on a surface until the corresponding contact angle increases to *θ*_*adv*_ or decreases to *θ*_*rec*_.

**Figure 2:**
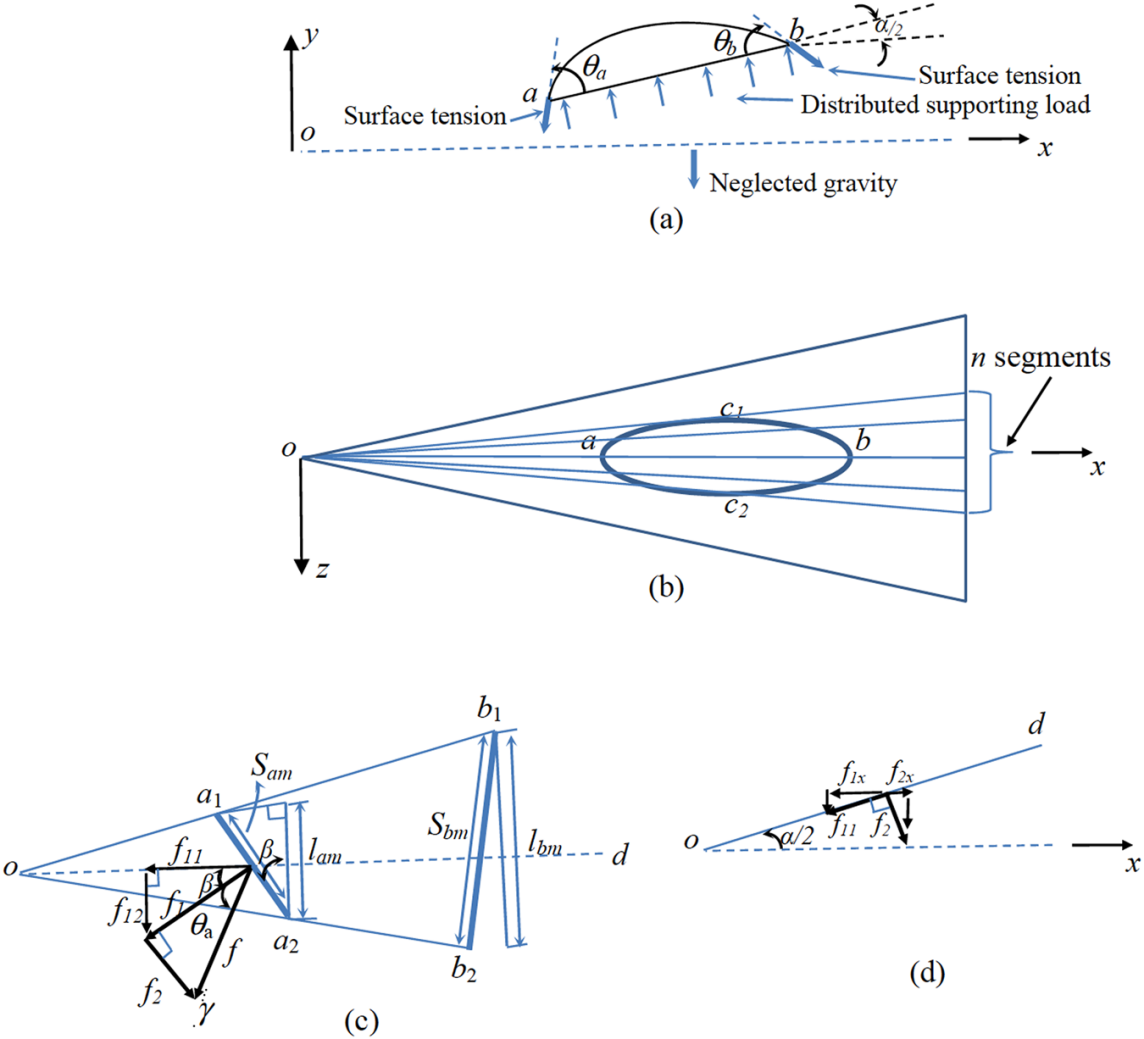
(**a**) Forces applied on a clam-shell drop (cross-sectional view). (**b**) Part of a wire surface that a clam-shell drop occupies is divided into *n* segments (top view). (**c**) Surface tension-induced force applied on a segment *a*_1_ *a*_2_ of the contact line *c*_1_ *ac*_2_ (perspective view), and (**d**) the components of surface tension-induced force on the plane, which is perpendicular to the segment and formed by *od* and *x* axis (side view).

A clam-shell drop at rest suffers three forces: gravity, supporting force of the wire, and surface tension-induced force (Fig. 2a). The drops considered in this work are small. To be specific, half of its longitudinal size is less than capillary length of the liquid, which is about 2.7 mm for water. Gravity is thus neglected, and only the other two forces are considered. As illustrated in free-body diagram (Fig. 2a), surface tension pulls a drop against the wire surface. The supporting force that the drop experiences is actually the reaction force exerted by the wire surface to balance the surface tension-induced force. This supporting force is a collection of supporting loads distributed at the interface of the drop and wire. These loads are perpendicular to the interface (Fig. 2a). Let *F*_*s*_ denote the resultant force of the distributed supporting loads. Due to the symmetry of the load distribution with respect to *x*-*y* plane, the *z* component of *F*_*s*_ is zero, the *x* component points to the negative direction of *x* axis, and *y* component is along the positive direction of *y* axis. Let *F*_*x*_ denote the magnitude of this *x* component. To determine *F*_*x*_, we have to figure out the distributed supporting loads. At the local area of the interface, the supporting loads equal liquid pressures applied at the other side of the interface. As discussed in the introduction session, the determination of these liquid pressures involves numerical calculation of the drop profile. Therefore, to simplify the problem, we choose not to determine *F*_*x*_.

Considering the balance of forces on a drop along the positive direction of *x* axis (Fig. 2a), we have

1 $$F={F}_{T}-{F}_{x},$$

where *F* represents the corresponding resultant force, and *F*_*T*_ denotes the *x* component of surface tension-induced force. To make a drop move towards the root, we should have

2 $$F > 0$$

for any allowed *θ*_*a*_ and *θ*_*b*_. Similarly, to ensure that a drop transports towards the tip, we should get

3 $$F < 0.$$

By Eq. (1), if Ineq. (2) holds true, where “Ineq.” is abbreviation of “Inequality”, then we have *F*_*T*_ > *F*_*x*_ > 0, which mean

4 $${F}_{T} > 0.$$

However, the converse is not true. Hence, Ineq. (4) is a necessary condition of Ineq. (2). Meanwhile, once

5 $${F}_{T} < 0,$$

Ineq. (3) is met, while the converse also does not hold true. Thus, Ineq. (5) is a sufficient condition of Ineq. (3). Based on the above discussions, Ineq. (4) is actually a necessary condition for a drop to move towards the tip, while Ineq. (5) is a sufficient one for the drop to transport towards the root.

### Surface tension-induced force on a clam-shell drop

As shown in Fig. 2(b), to find the expression of *F*_*T*_, we divide the part of the wire surface, where the drop is located, into *n* identical segments. Each segment has a wedge-like shape with the tip located at *o*. Consider the *m*-th segment (Fig. 2c), where *m* ranges from 1 to *n*. Let *a*_1_ *a*_2_ and *b*_1_ *b*_2_, respectively, denote the portions of *c*_1_ *ac*_2_ and *c*_2_ *bc*_1_ located inside this segment. Use *S*_*am*_ and *S*_*bm*_ to represent their arc lengths, respectively. Set *S*_*c*1*ac*2_ and ${S}_{{c}_{2}b{c}_{1}}$ to be arc lengths of *c*_1_ *ac*_2_ and *c*_2_ *bc*_1_, separately. Accordingly, we have

6 $${S}_{{c}_{1}a{c}_{2}}=\sum _{m=1}^{n}{S}_{am},$$

7 $${S}_{{c}_{2}b{c}_{1}}=\sum _{m=1}^{n}{S}_{bm}.$$

Without loss of generality, we consider the surface tension-induced force on *a*_1_ *a*_2_. The same line of reasoning also applies to *b*_1_ *b*_2_ in finding the corresponding surface tension-induced force applied over there.

Set *od* to be the middle line of the segment (Fig. 2c). Although this segment is curved, due to its small lateral size, it may be approximated as a local tangent plane to the wire surface along *od*. Subsequently, the plane, which is formed by *od* and *x* axis, is perpendicular to the segment. The surface tension-induced force, *f*, that is applied on *a*_1_ *a*_2_ is *γS*_*am*_. It can be decomposed into two components. The first component, *f*_1_, lies on the segment. The second one, *f*_2_, is perpendicular to this segment. Accordingly, it is parallel to the plane constructed by *od* and *x* axis. By geometric analysis, we have *f*_1_ = *γS*_*am*_cos*θ*_*a*_ and *f*_2_ = *γS*_*am*_sin*θ*_*a*_. The force component *f*_1_ can be further decomposed into *f*_11_ and *f*_12_ (Fig. 2c). *f*_11_ is along the direction of *od*. We get *f*_11_ = *γS*_*am*_cos*θ*_*a*_cos*β* = *γl*_*am*_cos*θ*_*a*_, where *β* is an angle defined in Fig. 2(c) and *l*_*am*_ denotes the length of the line segment that the chord of *a*_1_ *a*_2_ is projected on the plane perpendicular to *od*. Meanwhile, *f*_12_ is perpendicular to the plane formed by *od* and *x* axis. Accordingly, only *f*_11_ and *f*_2_ have components along *x* axis. By geometric analysis (Fig. 2d), we determine these force components as: *f*_1*x*_ = $-\gamma {l}_{am}\,\cos \,{\theta }_{a}\,\cos \,\frac{\alpha }{2}$ and *f*_2*x*_ = $\gamma {S}_{am}\,\sin \,{\theta }_{a}\,\sin \,\frac{\alpha }{2}.$ Thus, the *x*-component of the surface tension-induced force applied at *a*_1_ *a*_2_ is: $-\gamma {l}_{am}\,\cos \,{\theta }_{a}\,\cos \,\frac{\alpha }{2}+\gamma {S}_{am}\,\sin \,{\theta }_{a}\,\sin \,\frac{\alpha }{2}.$ Likewise, we found that the *x*-component of the surface tension-induced force applied at *b*_1_ *b*_2_ is:$\gamma {l}_{bm}\,\cos \,{\theta }_{b}\,\cos \,\frac{\alpha }{2}+\gamma {S}_{bm}\,\sin \,{\theta }_{b}\,\sin \,\frac{\alpha }{2}.$ Consequently, with the aid of Eqs. (6) and (7), the summation of the *x* components of such forces on all the segments leads to

8 $${F}_{T}=-\gamma \,\cos \,{\theta }_{a}\,\cos \,\frac{\alpha }{2}\sum _{m=1}^{n}{l}_{am}+\gamma {S}_{{c}_{1}a{c}_{2}}\,\sin \,{\theta }_{a}\,\sin \,\frac{\alpha }{2}+\gamma \,\cos \,{\theta }_{b}\,\cos \,\frac{\alpha }{2}\sum _{m=1}^{n}{l}_{bm}+\gamma {S}_{{c}_{2}b{c}_{1}}\,\sin \,{\theta }_{b}\,\sin \,\frac{\alpha }{2}.$$

In deriving *f*_1*x*_, *S*_*am*_ has been approximated as the chord length of *a*_1_ *a*_2_, whose projection is *l*_*am*_ on the plane perpendicular to *od*. Similar approximation was also made to find the *x*-component of the surface tension-induced force applied at *b*_1_ *b*_2_. These approximations brought an error to Eq. (8). According to ref. 18 (see its Section 4.1), the maximum possible error is in the order of $\frac{\gamma {S}^{2}}{{n}^{2}},$ where *S* denotes the arc length of the loop *c*_1_ *ac*_2_ *b*. This result indicates that the error of Eq. (8) decreases with the increase in the number of partitions.

Furthermore, by geometric analysis (Fig. 2c), it is readily shown that

9 $${l}_{bm} > {l}_{am},$$

10 $${S}_{{c}_{1}a{c}_{2}}\ge \sum _{m=1}^{n}{l}_{am},$$

11 $${S}_{{c}_{2}b{c}_{1}}\ge \sum _{m=1}^{n}{l}_{bm}.$$

### Surface tension-induced force on a barrel drop

Although the above analysis was initially developed for a clam-shell drop, it applies to a barrel drop as well, since this analysis is not limited by the drop profile. That is, Ineqs. (4) and (5) are also applicable to the barrel drop for judging its moving direction. Likewise, Eq. (8) also holds true for a barrel drop.

The barrel drop surrounds a conical wire. Hence, the whole wire surface is divided into small segments (Fig. S1(a)). Since this drop is axisymmetric with respect to *x* axis, *l*_*am*_ and *l*_*bm*_ become the chord lengths of *a*_1_ *a*_2_ and *b*_1_ *b*_2_, respectively (Fig. S1(b)). Consequently, for a large *n*, we approximately get

12 $$\sum _{m=1}^{n}{l}_{am}={S}_{{c}_{1}a{c}_{2}},$$

13 $$\sum _{m=1}^{n}{l}_{bm}={S}_{{c}_{2}b{c}_{1}}.$$

With the aid of these two equations, Eq. (8) is reduced to

14 $${F}_{T}=-\gamma {S}_{{c}_{1}a{c}_{2}}\,\cos \,({\theta }_{a}+\frac{\alpha }{2})+\gamma {S}_{{c}_{2}b{c}_{1}}\,\cos \,({\theta }_{b}-\frac{\alpha }{2}).$$

Comparison of Eq. (14) with Eq. (8) indicates that, because the barrel drop forms simple contact lines with the conical wire, which are two circles, the expression of *F*_*T*_ is much simplified.

### Moving conditions of a drop with no contact angle hysteresis

To give a sense about conditions for a drop to move on a conical wire, we first consider a specific situation. Two assumptions are made in this situation: the conical wire has a small conical angle, and contact angle hysteresis is 0°. Accordingly, the following two relations are satisfied:

15 $$\alpha \to {0}^{{\rm{o}}},$$

16 $${\theta }_{rec}={\theta }_{adv}=\theta ,$$

where *θ* denotes the unique contact angle of a drop on the wire. By Relation (15), we have

17 $$\sin \,\frac{\alpha }{2}=0,$$

18 $$\cos \,\frac{\alpha }{2}=1.$$

With the aid of Eqs. (16)–(18), Eq. (8) is reduced to

19 $${F}_{T}=\gamma \,\cos \,\theta (\sum _{m=1}^{n}{l}_{bm}-\sum _{m=1}^{n}{l}_{am}).$$

Subsequently, with the assistance of Ineq. (9), we find: i) if $\theta  < \frac{\pi }{2},$ then the right-hand side of Eq. (19) is larger than zero, which means that Ineq. (4) is satisfied; and ii) when $\theta  > \frac{\pi }{2},$ it is less than zero, which implies that Ineq. (5) holds true. These results indicate that, if contact angle hysteresis is zero, and if conical angles are also small, then liquid drops may move towards the root and tip, respectively, on lyophilic and lyophobic wires.

Seven related examples were simulated in ref. 10 (see its Figs 4 and 5). Three of them were regarding barrel drops, while the other four were about clam-shell drops. In these examples, receding and advancing contact angles were equal, and conical angles were less than 5°. Thus, the two assumptions of the specific situation were satisfied in each example. In six out of the seven examples, the contact angles were less than 90°. Accordingly, as predicted, the simulated drops moved towards the roots. In the remaining example, the contact angle was 150°. Consequently, the corresponding drop should move from the root towards the tip, which agrees with the corresponding simulation result of ref. 10.

In addition, in an experiment of ref. 7 (see its Fig. 1), a barrel drop of silicone oil was placed on a copper conical wire. In this example, the two assumptions of the specific situation were met as well, since receding and advancing contact angles of the silicone oil drop were both 0° on the copper conical wire. Thus, this drop is expected to move from the tip towards the root of the corresponding conical wire, which agrees with what was reported in ref. 7.

### Moving conditions of a drop with contact angle hysteresis

In practice, since the surface of a conical wire, such as those of cactus spines, is not ideally smooth, contact angle hysteresis may not be zero. Also, conical angles are not necessarily small. Hence, we further consider moving conditions in a more general context, in which contact angle hysteresis is not required to be zero and conical angles not small. These conditions are grouped into four cases, which are summarized in Table 1.

**Table 1:** Summary of the four cases.

	Relation (15)	Relations (20) and (21)	Relation (25)	Relation (26)	Relation (27)	Relation (28)	Moving situation
First case	Y	Y	N/A	N/A	N/A	N/A	From root towards tip
Second case	N/A	N/A	Y	Y	N/A	N/A	From tip towards root
Third Case	N/A	N/A	Y	N	Y	N/A	From tip towards root
Fourth case	Y	N/A	N/A	N/A	N/A	Y	Do not move from tip towards root

Y: Relation(s) satisfied. N: Relation(s) not met. N/A: Not applicable.

### Conditions for a drop to move towards the tip

We consider one case, which is also the first case of this work when contact angle hysteresis is not zero. In this case, we assume that Relation (15) and the following two relations

20 $${\theta }_{rec} > \frac{\pi }{2},$$

21 $$\frac{\sum _{m=1}^{n}{l}_{bm}}{\sum _{m=1}^{n}{l}_{am}} > \frac{\cos \,{\theta }_{adv}}{\cos \,{\theta }_{rec}}$$

hold true. As indicated in the previous session, in terms of Relation (15), Eqs. (17) and (18) are satisfied. Subsequently, it follows from Eq. (8) that

22 $${F}_{T}=\gamma \,\cos \,{\theta }_{b}\sum _{m=1}^{n}{l}_{bm}-\gamma \,\cos \,{\theta }_{a}\sum _{m=1}^{n}{l}_{am}.$$

With the aid of Ineqs. (22) and (9), as well as Relations (20) and (21), it is readily shown that Ineq. (5) is satisfied.

Ineq. (21) actually gives a lower limit to $\frac{\sum _{m=1}^{n}{l}_{bm}}{\sum _{m=1}^{n}{l}_{am}}$. This limit is $\frac{\cos \,{\theta }_{adv}}{\cos \,{\theta }_{rec}}$. To make Ineq. (21) hold true, a practical method is to increase drop size, which increases *l*_*bm*_ while decreases *l*_*am*_. In addition, when a drop is located closer to the tip, it is easier to make Ineq. (21) satisfied. The contact area of a drop with the wire surface should increase with the decrease in its distance to the tip, since the diameter of the conical wire decreases with this distance. Consequently, the difference between *l*_*bm*_ and *l*_*am*_ should increase in the same manner. In other words, once a drop satisfies Ineq. (21), it should still meet this inequality when it moves closer to the tip.

The results of this case indicate that, if the conical angle is small, and if a conical wire is also lyophobic, then a large drop should move towards the tip of this conical wire. The moving condition on a lyophobic wire in the specific situation that the contact angle hysteresis is zero actually belongs to this case. Another example of the case is that there exists a wetting gradient from the root to the tip of a lyophobic wire. That is, the contact angle decreases along the direction from the root to the tip. In this example, *θ*_*rec*_ in Ineq. (21) refers to the minimum possible value of *θ*_*b*_, while *θ*_*adv*_ is the maximum possible value of *θ*_*a*_. Due to the wetting gradient, the difference between *θ*_*adv*_ and *θ*_*rec*_ is reduced. Accordingly, by Ineq. (9), Ineq. (21) may hold true even for a small drop. If ${\theta }_{adv}\le {\theta }_{rec}$, then a drop with any size should be able to move towards the tip on the corresponding wire. It was reported in ref. 19 that water drops moved away from the root towards the tip on a super-hydrophobic conical wire. The wetting degree on the wire surface increased along the same direction, which implies that the assumption of the aforementioned example was met. Thus, as expected, even small drops were observed in ref. 19 to move from the root towards the tip on the corresponding wire.

### Conditions for a drop to move towards the root

With the aid of Ineqs. (10) and (11), by Eq. (8), we have

23 $$\begin{array}{rcl}{F}_{T} & \ge  & -\gamma \sum _{m=1}^{n}{l}_{am}\,\cos \,{\theta }_{a}\,\cos \,\frac{\alpha }{2}+\gamma \sum _{m=1}^{n}{l}_{am}\,\sin \,{\theta }_{a}\,\sin \,\frac{\alpha }{2}\\  &  & +\gamma \sum _{m=1}^{n}{l}_{bm}\,\cos \,{\theta }_{b}\,\cos \,\frac{\alpha }{2}+\gamma \sum _{m=1}^{n}{l}_{bm}\,\sin \,{\theta }_{b}\,\sin \,\frac{\alpha }{2}.\end{array}$$

According to trigonometric relations, this inequality can be simplified as

24 $${F}_{T}\ge \gamma \sum _{m=1}^{n}{l}_{bm}\cos \,({\theta }_{b}-\frac{\alpha }{2})-\gamma \sum _{m=1}^{n}{l}_{am}\cos \,({\theta }_{a}+\frac{\alpha }{2}).$$

Exploration of Relation (24) results in two cases, which are referred to as the second and third cases of this work.

In the second case, we assume

25 $${\theta }_{adv} < (\frac{\pi }{2}+\frac{\alpha }{2}),$$

26 $$({\theta }_{adv}-{\theta }_{rec}) < \alpha .$$

According to these two inequalities, both $\cos ({\theta }_{b}-\frac{\alpha }{2}) > 0$ and $\cos ({\theta }_{b}-\frac{\alpha }{2}) > \,\cos ({\theta }_{a}+\frac{\alpha }{2})$ hold true for any allowed ${\theta }_{a}$ and ${\theta }_{b}$. Subsequently, with the aid of Ineq. (9), the right-hand side of Ineq. (24) is larger than zero. It then follows from Ineq. (24) that *F*_*T*_ > 0. That is, when Ineqs. (25) and (26) are met, Ineq. (4) is satisfied. This result implies that, if a conical wire is lyophilic, and if contact angle hysteresis is also less than conical angle, then a drop may move towards the root on this conical wire.

In the third case, we have three assumptions: Ineq. (25) is satisfied, Ineq. (26) is not, and the following inequality

27 $$\frac{\sum _{m=1}^{n}{l}_{bm}}{\sum _{m=1}^{n}{l}_{am}} > \frac{\cos \,({\theta }_{rec}+\frac{\alpha }{2})}{\cos \,({\theta }_{adv}-\frac{\alpha }{2})}$$

is met. Because Ineq. (26) is violated, we get $\cos ({\theta }_{adv}-\frac{\alpha }{2}) < \,\cos ({\theta }_{rec}+\frac{\alpha }{2})$. Since Ineq. (25) is met, we have $\cos ({\theta }_{adv}-\frac{\alpha }{2}) > 0.$ Subsequently, although $\cos \,({\theta }_{adv}-\frac{\alpha }{2}) < \,\cos \,({\theta }_{rec}+\frac{\alpha }{2})$, by Ineq. (27), we still obtain $[\sum _{m=1}^{n}{l}_{bm}\,\cos \,({\theta }_{adv}-\frac{\alpha }{2})-\sum _{m=1}^{n}{l}_{am}\cos \,({\theta }_{rec}+\frac{\alpha }{2})] > 0$. Accordingly, it follows from Ineq. (24) that *F*_*T*_ > 0. Hence, the corresponding drop may run towards the root.

Ineq. (27) also gives a lower limit to $\frac{\sum _{m=1}^{n}{l}_{bm}}{\sum _{m=1}^{n}{l}_{am}}$, which is $\frac{\cos \,({\theta }_{rec}+\frac{\alpha }{2})}{\cos \,{\theta }_{adv}-\frac{\alpha }{2}}$. As in the case of Ineq. (21), to make Ineq. (27) hold true, a practical approach is to increase drop size. Also, it is easy to satisfy Ineq. (27) when a drop is close to the tip. However, when the drop transports a certain distance away from the tip, it may stop due to the violation of this inequality.

In summary, the result of this case indicates that, if contact angle hysteresis is larger than conical angle, and if a conical wire is also lyophilic, then a large drop may still move towards the root on the conical wire. Also, it is possible that a drop stops after it moves a certain distance towards the root.

The moving condition on a lyophilic wire in the specific situation that the contact angle hysteresis is zero belongs to the second case. In addition, although drops were reported in refs 1–3, 14, 20–22 to move from tips towards roots, their results could not be used to examine the second and third cases, since the receding and advancing contact angles in the corresponding tests were not explicitly given. In ref. 15 (see its Fig. S2), receding and advancing contact angles were measured during the motion of a drop. These contact angles were less than 90°. Meanwhile, Ineq. (26) was met at some spots of a cactus spine, but not at the other locations. Accordingly, the moving situation is predicted to be a combination of the second and third cases. This prediction matches what was observed from Video S1 of ref. 15.

### Conditions for a drop not to move towards the root

Further consideration of Eq. (8) leads to the fourth case of this work. In this case, we assume that both Ineq. (15) and the following inequality are satisfied:

28 $${\theta }_{adv} > \frac{\pi }{2}.$$

As demonstrated in the first case, by Ineq. (15), Eq. (22) is also met. Subsequently, with the aid of Eq. (22) and Ineqs. (9) and (28), it is readily shown that Ineq. (5) is violated, which means that the corresponding liquid drop cannot move towards the root.

In this case, receding contact angle can be either less or larger than $\frac{\pi }{2}$. The result of this case not only agrees with that of ref. 8, but also indicates that, no matter where a drop is initially located and whether it has a barrel or clam-shell shape, this drop cannot move towards the root of a lyophobic conical wire with small conical angle.

## Experimental design

**Figure 3:**
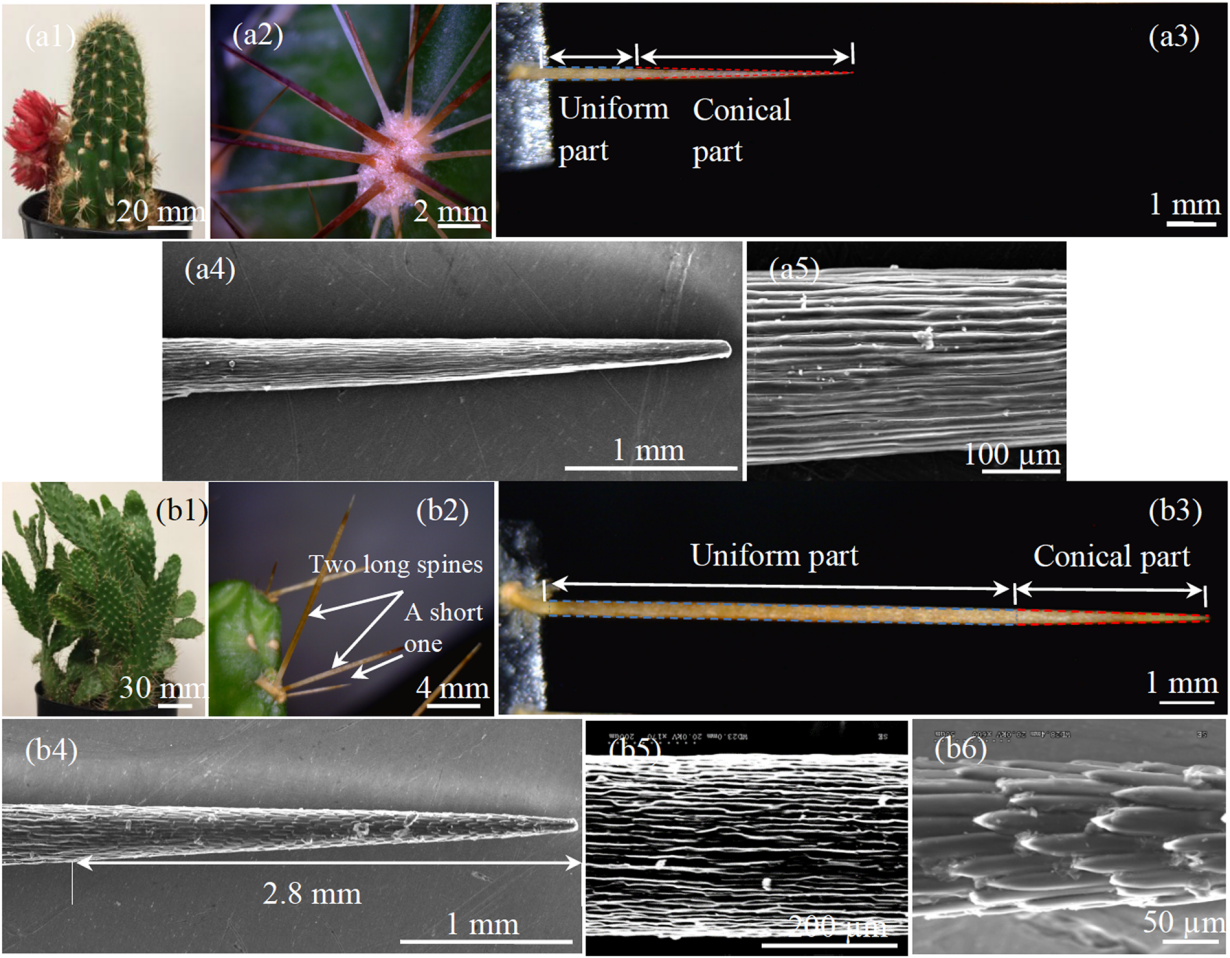
(a1) Perspective view of cactus *Torch*, whose surface is distributed with an array of clusters, (a2) 3-D view of a cluster with 18 spines, and (a3) side view of a 7-mm-long spine, whose uniform part is 2.2 mm long, conical part is 4.8 mm long, and conical angle is 4°. (a4) Scanning electron microscope (SEM) image of a single spine, and (a5) close-up view of microgrooves that are located on the spine surface. (b1) Perspective view of cactus *Consolea falcate*, whose surface is also distributed with an array of clusters, (b2) 3-D view of a cluster with one short and two long spines, and (b3) side view of a 16-mm-long spine, whose uniform part is 11.3 mm long, conical part is 4.7 mm long, and conical angle is 3°. (b4) SEM image of the 2.8-mm-long tip portion that is covered by microbarbs. Microgrooves are seen on the non-tip portion. Close-up views of (b5) microgrooves and (b6) microbarbs.

Some experimental or numerical examples have been found from literature to validate our derived conditions. Meanwhile, water drops have been previously reported to self-run from tips towards the roots on the spines of some cactus species^1, 14, 15^. This directional movement is related to our second to fourth cases. To have a better understanding about the behavior of drops on cactus spines, as well as to provide more experimental results for examining the second to fourth cases, in this work we did tests on spines of two cactus species: *Torch* and *Consolea falcate* (Fig. 3). A spine of either cactus includes two portions: conical and uniform. The conical portions of the *Torch* spines that are tested have the lengths between 4.8 and 6.2 mm. Their conical angles range from 3° to 4° with a measurement error of 0.5°. The uniform portions vary from 2.2 to 2.8 mm in their lengths. They have almost identical cross-sections with the diameters of about 300 μm. The *Consolea falcate* spines that are tested have lengths in the range of 16 to 22 mm. The conical part of a tested spine is about 4.7 to 7.5 mm long. It also has a conical angle in the range of 3° to 4°. The uniform portion of a tested spine has a length varying from 11.3 to 14.5 mm with a diameter of around 330 μm.

Three groups of experiments are done on four spine samples: *Torch* spines without any additional surface treatment, *Consolea falcate* spines without any additional surface treatment, Teflon-coated *Torch* spines, and Teflon-coated *Consolea falcate* spines. In the first group of experiments, single drops of four liquids are tested on the spine samples to examine the second through fourth cases. The four liquids are water, isopropyl alcohol (IPA), silicone oil, and water/IPA mixture. The mixture of water and silicone oil is not tested, since these two liquids are immiscible. Water and IPA are miscible. Furthermore, their mixtures with various weight ratios have different contact angles on spine samples, providing more experimental data to validate the derived conditions. Consequently, their mixtures are examined in this work. The corresponding contact angles are measured using an approach similar to that of refs 23 and 24 (Table 2). The second group of experiments simulates the situation of a fog test, which has small water drops emerge at the beginning of the test. On the other hand, they differ in the way that water is supplied. In the second group of experiments, additional water drops are manually added to the small ones. However, in the fog test, water drops come from the mist flow. In addition, in comparison with the first group of experiments, the second group also provides a chance to examine the behaviors of single water drops at different spots of a spine sample. The manually supplied drops in our experiments have diameters in the range of 0.5 to 1 mm.

**Table 2:** Contact angles of water, IPA and silicone oil measured on representative cactus spines, validated cases and corresponding type of drop movements. To avoid cross-contamination, different liquids were tested on different spine samples.

		*θ*_*rec*_(°)	*θ*_*adv*_(°)	$$\begin{array}{c}{\theta }_{adv}-{\theta }_{rec}\end{array}$$(°)	*α* (°)	Validated case	Type of movements
Untreated Spine of cactus *Torch*	100% Water	55	67	12	3	Third	Second
	75% Water and 25 % IPA	41	43	2	4	Second	First
	50% Water and 50 % IPA	39	41	2	3	Second	First
	25% Water and 75 % IPA	32	33	1	3	Second	First
	100% IPA	42	44	2	3	Second	First
	Silicone oil	40	42	2	4	Third	First
Teflon-coated spine of cactus *Torch*	100% Water	100	113	13	4	Fourth	Third
	75% Water and 25 % IPA	59	91	32	3	Third	Third
	50% Water and 50 % IPA	63	55	8	3	Third	Second
	25% Water and 75 % IPA	50	59	9	3	Third	Second
	100% IPA	43	50	7	4	Third	Second
	Silicone oil	42	52	10	3	Third	Second
Untreated spine of cactus *Consolea falcate*	100% Water	60	97	37	4	Fourth	Third
	75% Water and 25 % IPA	34	36	2	4	Second	First
	50% Water and 50 % IPA	34	36	2	4	Second	First
	25% Water and 75 % IPA	33	34	1	4	Second	First
	100% IPA	45	46	1	3	Second	First
	Silicone oil	37	38	1	3	Second	First
Teflon-coated spine of cactus *Consolea falcate*	100% Water	102	107	5	4	Fourth	Third
	75% Water and 25 % IPA	55	92	37	4	Fourth	Third
	50% Water and 50 % IPA	49	58	8	3	Third	Second
	25% Water and 75 % IPA	47	55	8	4	Third	Second
	100% IPA	45	50	5	4	Third	Second
	Silicone oil	48	54	6	4	Third	Second

The second group of experiments includes two tests (Fig. S2). The first test has two operations. First, an array of water drops is placed on a spine sample. Second, a water drop is then added between the pair of drops located closest to the tip. The second operation is repeated multiple times. The second test also has two operations. These two operations are similar to the ones of the first test. The only difference is that, in the second operation of the second test, a water drop is added to the pair of drops that are located closest to the root, instead of the pair closest to the tip. In the two tests, water drops are supplied along the opposite directions to examine whether the direction of water supply affects that of drop movements.

In the third groups of experiments, fog tests are conducted on the spine samples. The cactus spines, measurement of contact angles, and experimental set up of the fog tests are detailed in Supplementary information (Figs S3 and S4).

## Experimental Results and Discussions

Three types of movements (Fig. 4) were observed from experimental tests (Figs 5–9, S5–S11). All the three types of movements were found in the first group of tests (Table 2, Fig. 5, S5 and S6), while the second and third types were seen in the second and third groups (Figs 6–9, S7–S11, and Supplementary Videos 1 and 2). Through the three groups of experiments, the second to fourth cases were each supported by at least nine testing results.

**Figure 4:**
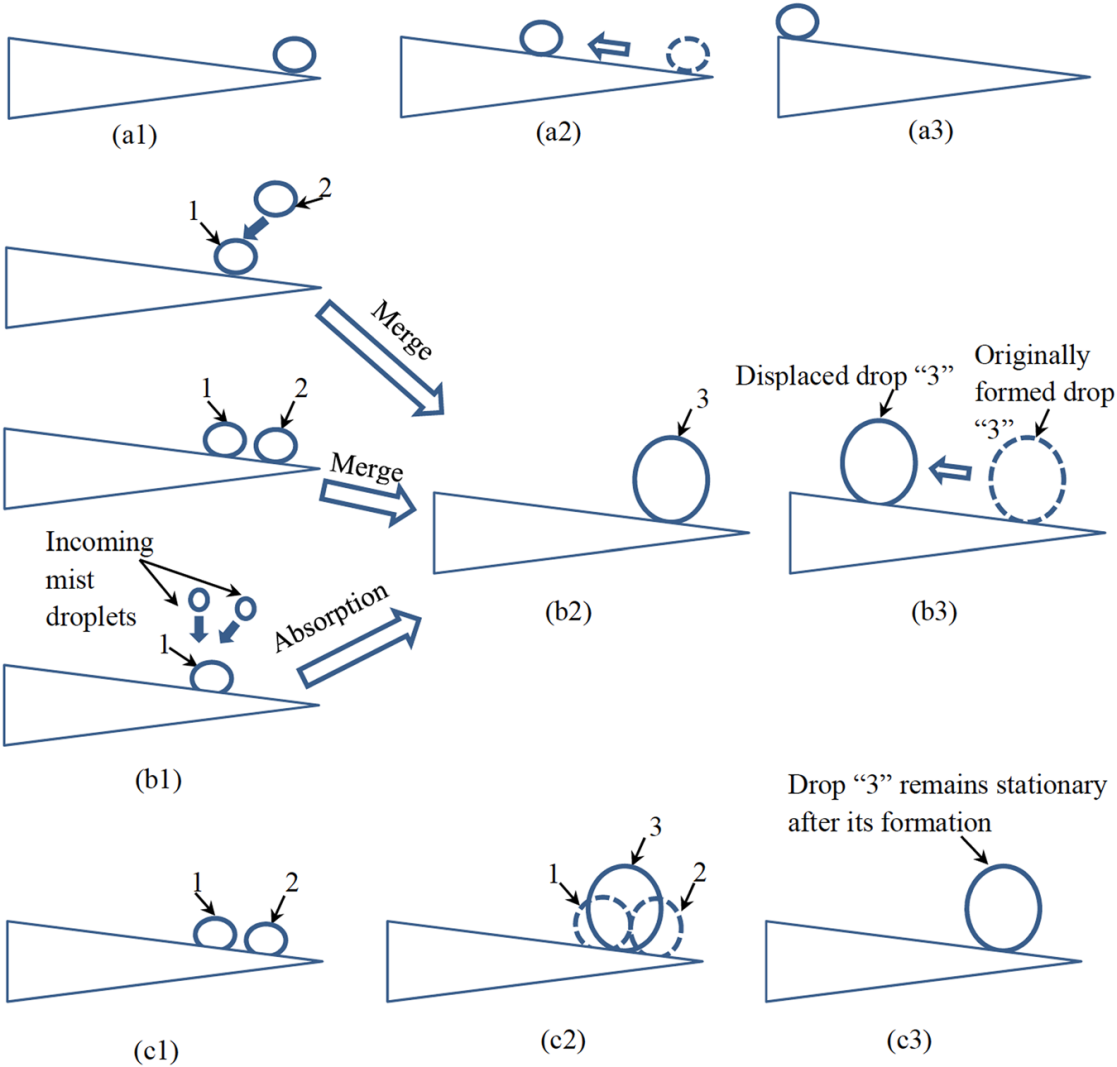
First type of drop movements: (a1) a drop is placed on the tip of a conical wire; (a2) it immediately moves towards the root; and (a3) the drop does not stop till it reaches the root. Second type of drop movements: (b1) drop “1” grows its size by merging with a manually added drop “2” or with its neighboring drop “2”, or by absorbing incoming mist droplets; (b2) the resulting large drop is called “3”; and (b3) drop “3” moves a certain distance towards the root and then stops. Third type of drop movements: (c1) two neighboring drops “1” and “2” are at rest; (c2) the two drops merge to form drop “3” due to addition of an additional drop or absorbing incoming mist droplets; and (c3) drop “3” remains stationary. Drop “3” self-runs towards the root relative to drops “1” and “2” in the second type of movements, while it just shifts slightly to the right and left relative to these two drops, respectively, in the third type.

**Figure 5:**
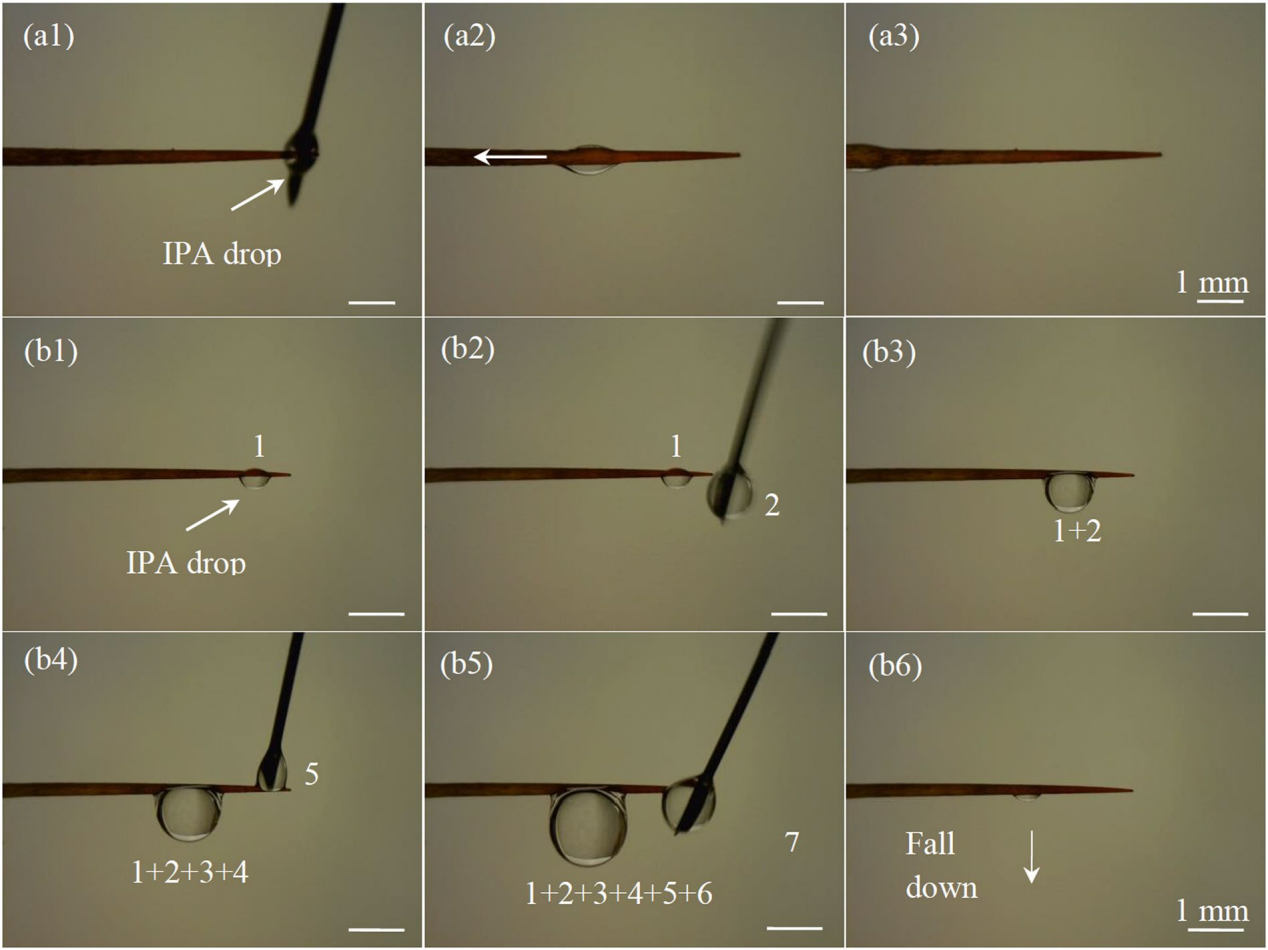
Transport of an IPA drop on an untreated *Torch* spine: (a1) the drop was released on the tip of an untreated *Torch* spine, (a2) it self-ran towards the root of the spine, and (a3) it finally stopped at the root. (b1)-(b5) Stop-and-go movement of IPA on a Teflon-coated *Torch* spine: continuous addition of new drops enabled the existing one to move towards the root step-by-step till it became stationary at the end of the conical part. (b6) Further addition of new drops made it then fall down. The motion observed in (a1–a3) belong to the first type of movements, while the one in (b1–b6) is the second type.

**Figure 6:**
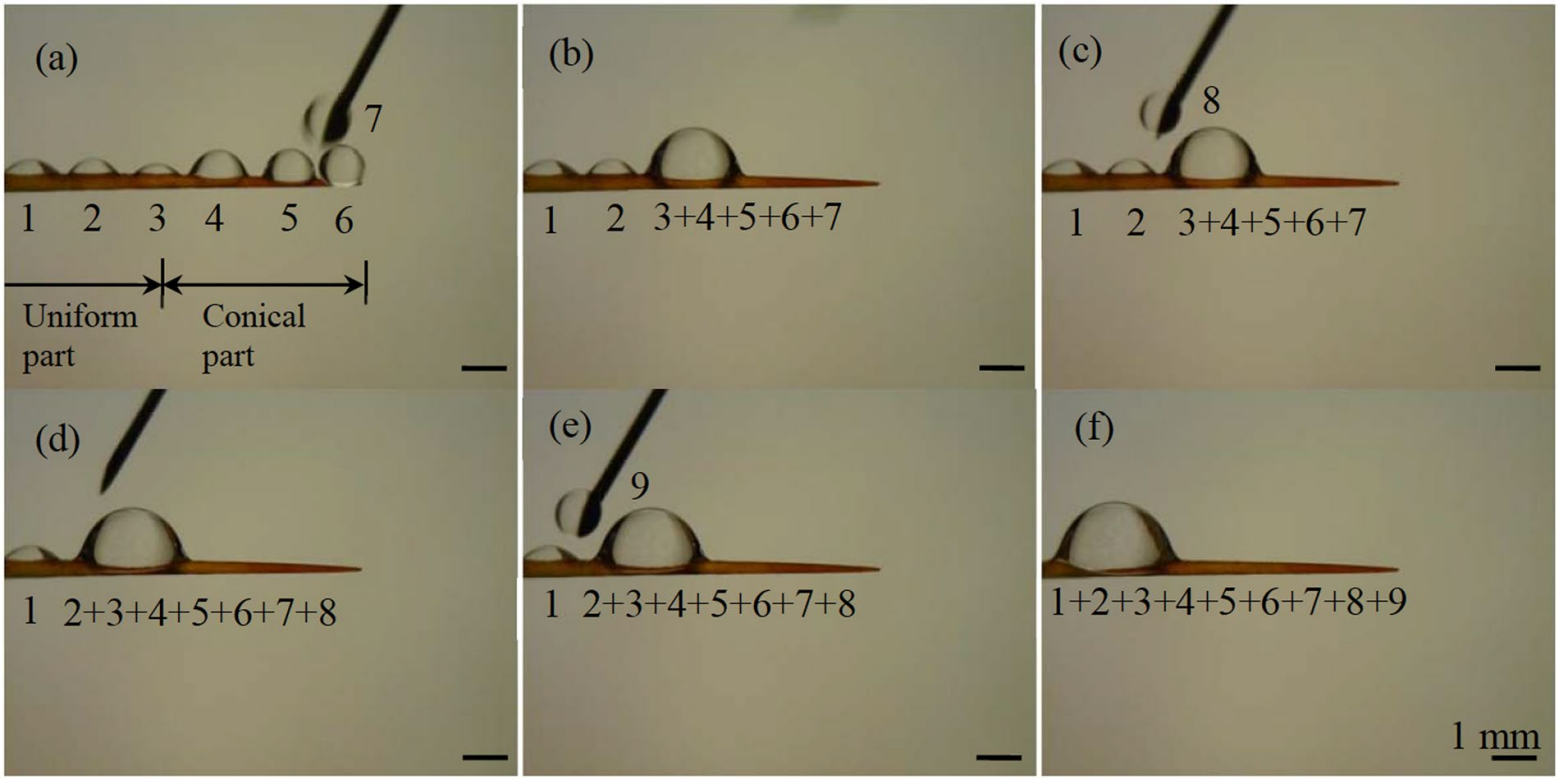
Representative results of the first test of the second group of experiments on an untreated *Torch* spine: (**a**) six water drops were first put on the spine surface, and drop “7” was added between drops “5” and “6”; (**b**) after this addition, the newly formed drop ran towards the root, merged with drops “4” and “3”, and then stopped at the end of the conical part; (**c**), (**d**) addition of drop “8” between drops “2” and “3+4+5+6+7” generated a new drop, which did not move beyond original location of drop “2”; and (**e**), (**f**) addition of drop “9” between drops “1” and “2 + 3 + 4 + 5 + 6 + 7” yielded a large drop, which also did not move beyond original location of drop “1”, completing this test. At the end of this test, all the drops merged into a large one on the uniform part of this spine, which was close to the root. The motion observed in (**a–f**) belongs to the second type of movements.

**Figure 7:**
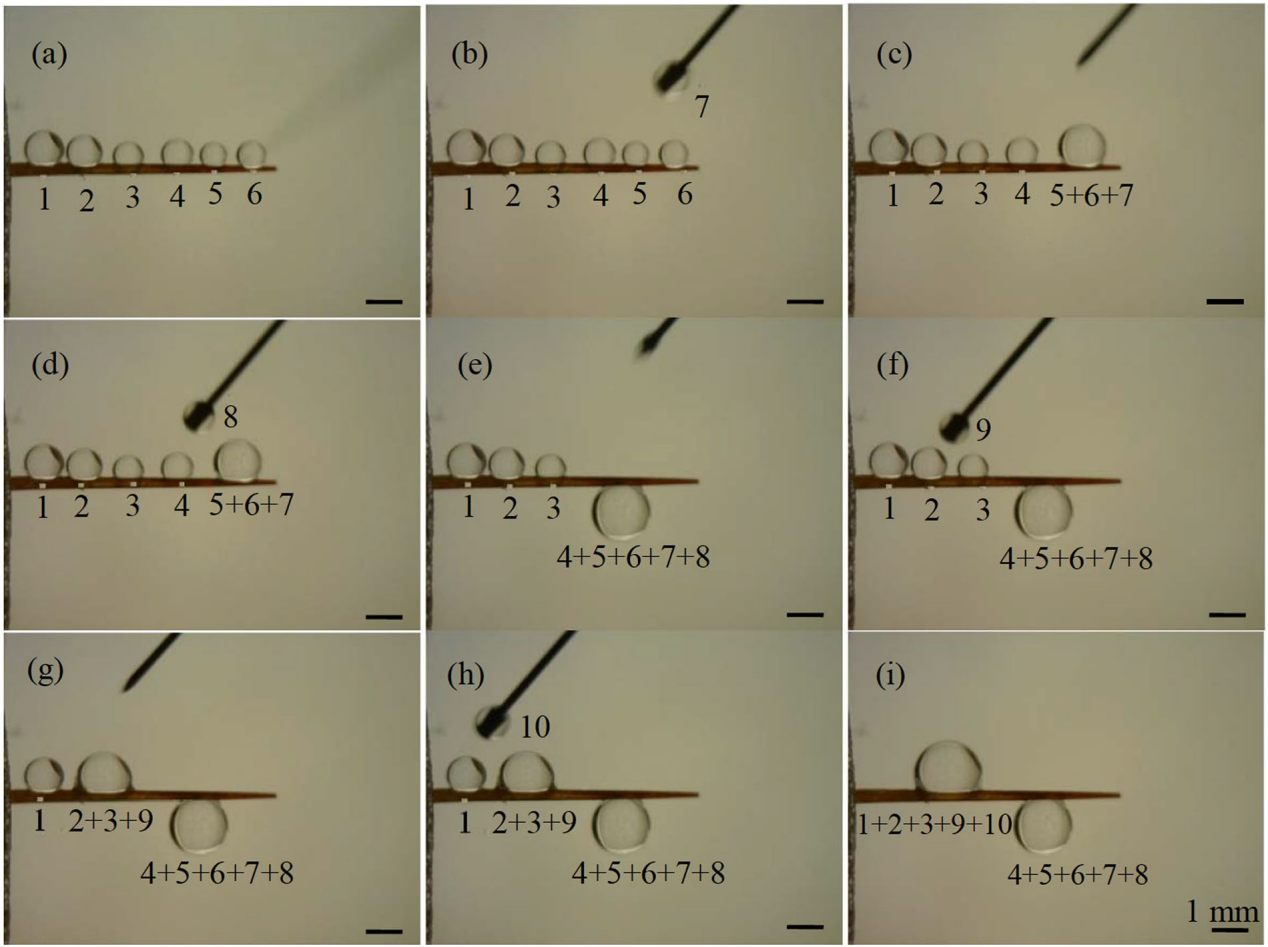
Representative results of the first test of the second group of experiments on a Teflon-coated *Torch* spine: (**a**) six water drops were first put on the spine surface; (**b–h**) after a new drop was put between a pair of two neighboring drops, a large drop was formed, which was stationary and located between the original positions of the pair of drops; and (**i**) two large drops were finally formed, respectively, in the middle portions of the uniform and straight parts of the spine. Further addition of a water drop between these two large drops resulted in a drop which subsequently fell down from the spine (image not shown). The motion observed in (**a–i**) belongs to the third type of movements.

**Figure 8:**
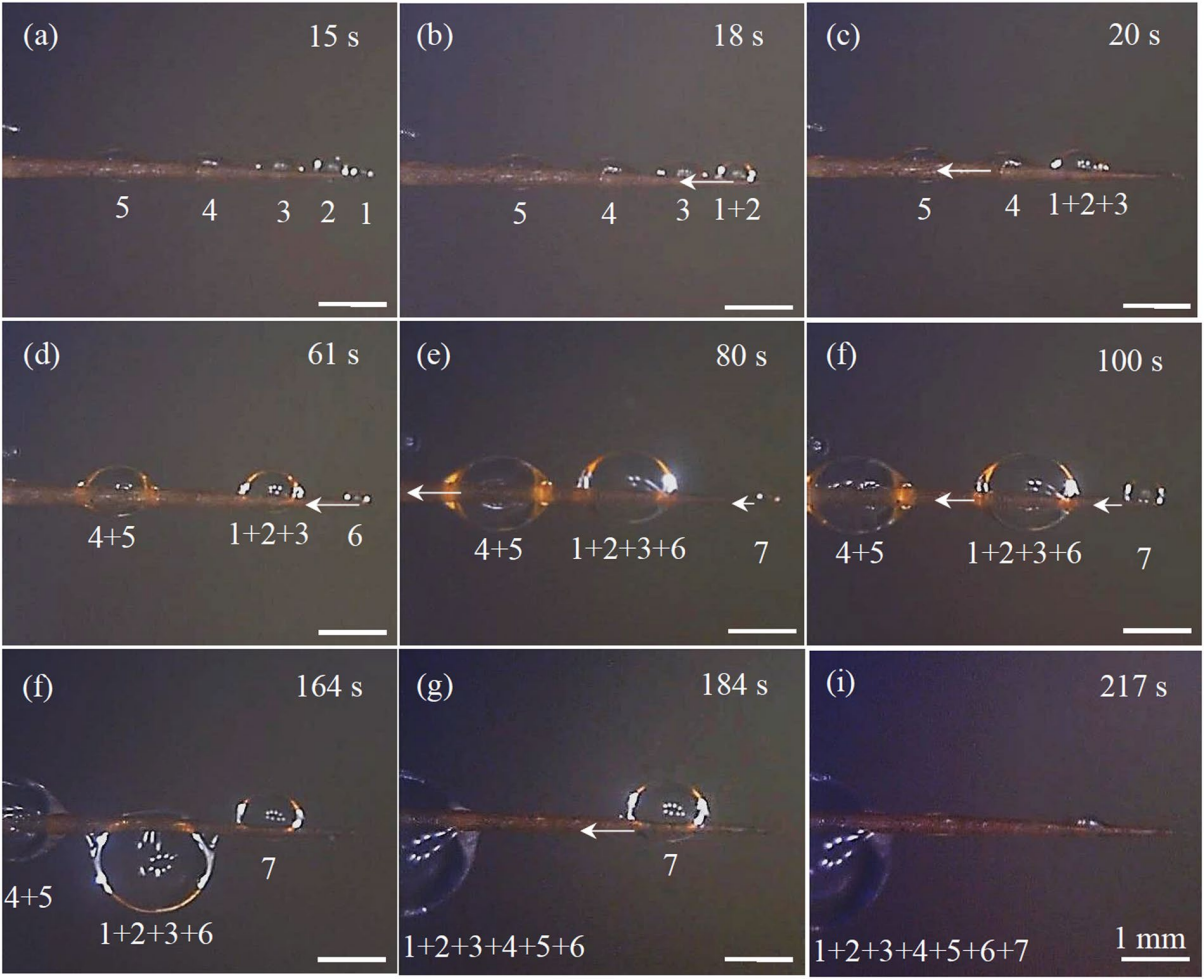
(**a–i**) Water drop movements on an untreated *Torch* spine at different time instants of a fog test. The motion observed in (**a–i**) belongs to the second type of movements. The pictures of this figure were taken in a foggy environment. As such, they are not as clear as the ones shown in Figs 5–7, for which there was no mist flows around spine samples. The same applies to the images in Fig. 9, S10 and S11 that are also related to fog tests.

**Figure 9:**
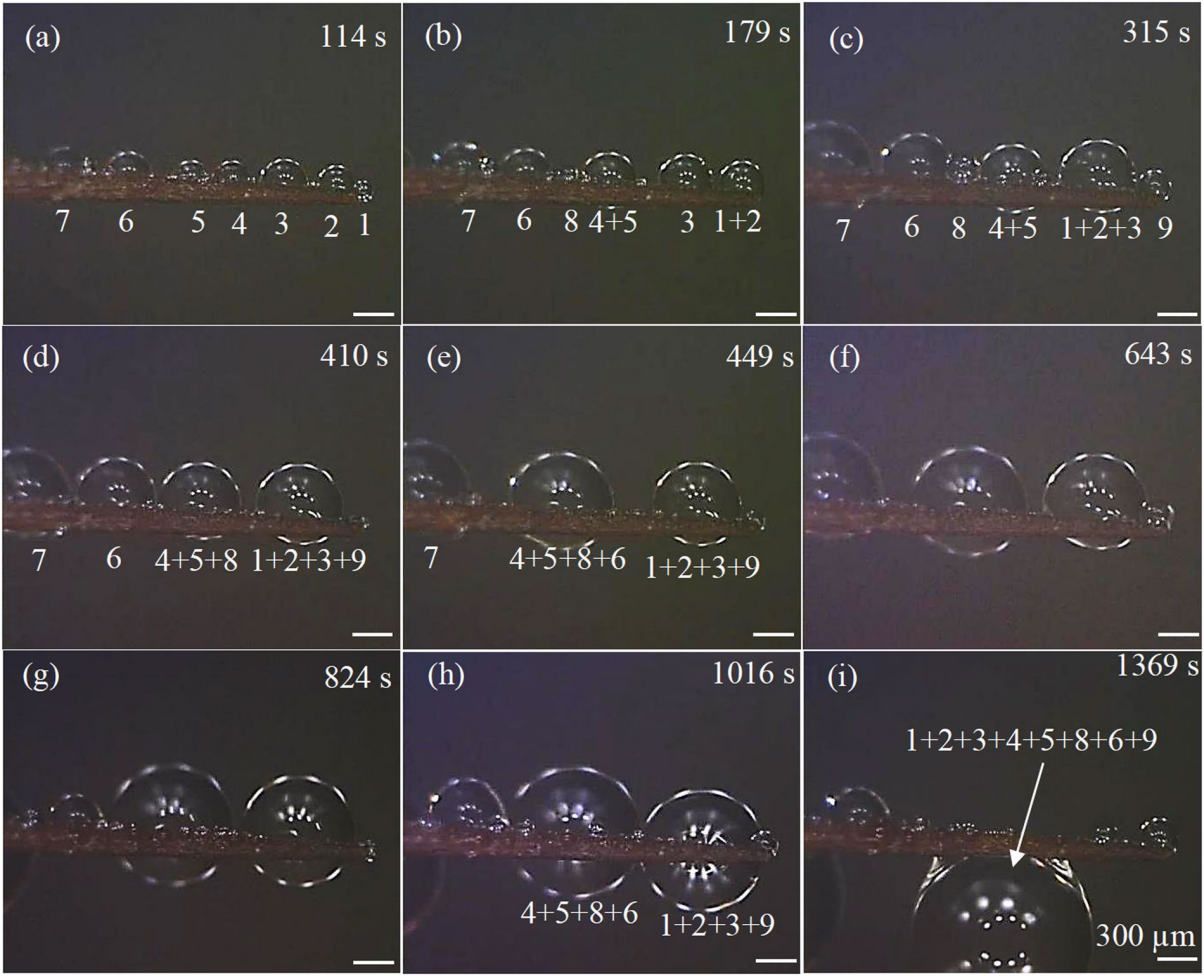
(**a–i**) Water drop movements on a Teflon-coated *Torch* spine during a fog test. The motion observed in (**a–f**) belongs to the third type of movements. The viewing area in this figure is smaller than that in Fig. 8, since the drops in Fig. 8 have a longer moving range. Accordingly, scale bars in this figure represent smaller sizes.

In the first type of movements, a liquid drop directly moved from the tip to the root of a spine sample (Fig. 4a). As observed from Table 2, the two untreated spine samples were lyophilic to both IPA and oil. The contact angle hysteresis of either IPA or oil was less than the corresponding conical angle on these spine samples. Accordingly, the two assumptions of the second case were satisfied on the untreated spines of both cacti. The same also applied to water/IPA mixture, when the weight concentration of water in water/IPA mixture was 75% or below (Table 2). Hence, as expected, the drops of the corresponding liquids had the first type of movements on these two spine samples (Fig. 5a, S5 and S6). It is known that IPA is easy to evaporate. In our tests, it took at least 90 s for a mm-scaled IPA drop to completely evaporate, while no more than 3 s for the drop to finish its motion on a cactus spine. Thus, the effect of evaporation was negligible.

In the second type of movements, liquid drops had stop-and-go motions (Figs 4 b, 5 b, 6, 8 and S8). For example, as shown in Fig. 5(b), an IPA drop stopped after travelling a short distance towards the root. It moved again after its drop size was increased. When this type of movements occurred, contact angle hysteresis of a liquid drop on the corresponding spine sample was larger than conical angle, and the spine sample was lyophilic to the liquid drop (Table 2). That is, the two assumptions of the third case were satisfied. Thus, as expected from the results of this case, the liquid drop may have stop-and-go motions.

In the third type of movements, a drop changed its position by merging with another drop (Fig. 4c). The resulting large drop was stationary, and it just shifted slightly to the right and left relative to the pair of small drops that formed it. In the second and third groups of experiments, the second type of movements occurred on untreated *Torch* samples (Figs 6, S8 and 8, and Supplementary Video 1), while the third type of movements happened on the other three spine samples (Figs 7, 9, S7, S9–S11, and Supplementary Video 2).

The third type of movements may occur due to two reasons:

I). Ineq. (27), which is one of the three assumptions for the first case, appears difficult to satisfy. This may be the reason why water drops did not move towards the tip in the third type of movements. In principle, increasing a drop size to a certain degree should make this inequality satisfied. However, in practice, this did not work as expected. As observed from the two tests on Teflon-coated spines of both cacti, a drop with a diameter of 2 mm was still stationary on the conical part of a spine (Fig. 7i). Addition of a 1 mm drop might cause this drop to fall down from the spine, due to two factors: a dynamic force generated during the merging process or produced in the procedure that a drop is put on a spine, and small contact area between a drop and a hydrophobic spine. In our derivation of the first case, we did not consider these two factors, which will be left for future investigation. In the case of ref. 19, water drops adhered to the surface of a super-hydrophobic conical wire due to the incorporation of nanohairs on the wire surface, providing an approach to avoid the falling of water drops from a hydrophobic wire. As previously discussed, the water drops self-ran towards the tip of the corresponding super-hydrophobic wire^19^, supplying an experimental evidence to support the first case.

II). Because the assumption of the fourth case was satisfied on the untreated *Consolea falcate*, Teflon-coated *Consolea falcate* and Teflon-coated *Torch* samples, even a large water drop could not self-run towards the root in the third type of movements.

The second and third types of movements are, respectively, similar to those observed on hydrophilic and hydrophobic copper conical wires^9^. In addition, the second type of drop movements was also seen on spines of other cacti species^1, 14, 15^, spider silks^2^, water striders’ legs^3^, PI fibers-covered silver needles^20^, and barbs of barley (*Hordeum vulgare*) awns^21^. The third type was observed on bare silver needles^20^. In addition, in the two tests of the second group of experiments, the direction of water supply did not change the type of drop movements that occurred on a spine sample. A critical difference between the second and third types of movements is (Fig. 4(b3) and (c3)): a drop in the second type may self-run towards the root of a cactus spine, while the one in the third type does not self-transport towards either the root or tip.

In the third group of tests, as a result of the second type of movements, all the small water drops that had appeared at the beginning of a fog test (Fig. 8a) moved to the root of the untreated *Torch* spine (Fig. 8f). In contrast, due to the third type of movements, almost all the small water drops, that had initially appeared on the other three spine samples, merged together to form large drops, which were located in the middle portion of the tested sample at the end of the fog tests if these drops did not fall off from the samples during the tests (Fig. 9i, S9 and S10). The second type of drop movements enables drops to transport to the root, while the third type may not. Hence, from the standpoint of fog collection, untreated *Torch* spines are capable of harvesting water from fog, while untreated *Consolea falcate* spines are not good options.

## Summary and Conclusions

In this work, we derived some conditions to judge whether a drop might move on a conical wire. These conditions apply to both barrel and clam-shell drops. Given that contact angle hysteresis is zero and that conical angle is small, drops may move towards the root and tip, respectively, on lyophilic and lyophobic wires. When these two constraints were relaxed, we found four cases: i) if conical angle is near 0° while the wire is lyophobic, then a large drop, which satisfies Ineq. (21), should also move towards the tip on a conical wire; ii) if contact angle hysteresis is less than conical angle, then a drop may move towards the root on a lyophilic conical wire; iii) if the former is larger than the latter, then this directional movement only happens to a large drop, which satisfies Ineq. (27); and iv) if conical angle is near 0° while advancing contact angle is larger than 90°, then a drop cannot move towards the root on the corresponding conical wire. The first case gives a sufficient condition for liquid drops to move towards the tips of conical wires, the second and third cases provide two independent conditions that may result in drop movements towards the roots, and the fourth gives a condition to judge whether a liquid drop moves towards the root of a conical wire. Ineqs. (21) and (27) are used to determine the minimum sizes that liquid drops should have to make the corresponding directional movements in the first and third cases, respectively. Due to lack of analytic expression of drop profiles, these two inequalities just give qualitative descriptions of the drop sizes, except in two special examples of the first case. Accordingly, the general models in the first and third cases may be considered to be qualitative. However, those in the other two cases are quantitative, since they are independent of drop sizes.

The derived conditions in the four cases have been validated using examples of literature and three groups of tests. In addition, in our previous consideration of a wire with small conical angle (see Eq. (12) of ref. 8), we found: a) the pressure difference inside a drop is inversely proportional to the conical angle, and b) this pressure difference increases with the decreases in the contact angle when the contact angle hysteresis is fixed. Conical wires have been employed in artificial fog collectors^9, 14, 20^ to collect fog. The results of our previous and current works provide the following two guidelines to develop artificial fog collectors for efficiently transporting both barrel and clam-shell drops: a) wire surface should be highly lyophilic, and b) conical angle should be as small as possible with contact angle hysteresis preferred to be less than this angle.

## Electronic supplementary material

Supplementary Information

Supplementary video 1

Supplementary video 2
