# Supplementary material for: Conditions for Barrel and Clam-Shell Liquid Drops to Move on Bio-inspired Conical Wires
Source: Sci Rep. 2017 Aug 29;7:9717. doi: 10.1038/s41598-017-10036-3 (PMC5575085; doi:10.1038/s41598-017-10036-3)
Supplement: Supplementary file 1 — Supplementary Information [file 41598_2017_10036_MOESM1_ESM.pdf]

Supplementary information

Conditions for Barrel and Clam-Shell Liquid Drops to Move on Bio-inspired Conical Wires

*Cheng Luo and Xiang Wang

Department of Mechanical and Aerospace Engineering, University of Texas at Arlington

500 West First Street, Woolf Hall 226, Arlington, TX 76019, the United States of America;

*email: chengluo@uta.edu

This supplementary information includes three parts: (i) descriptions of cactus spines, measurement of contact angles, and experimental set up of the fog tests; (ii) figures; and (iii) two videos corresponding to Figs. 6 and 7, respectively. Figures and references here are numbered according to the orders that they are cited in the main text.

Tested cactus spines

In this work we consider two cactus species: *Torch* and *Consolea falcate,* which were purchased from a local plant store (Figs. 3(a1) and 3(b1)). As in the cases of cacti *O. microdasys*[1], *Opuntiaengelmannii var. lindheimeri*[12] and *Gymnocalycium baldianum*[13], they also have an array of clusters on their pad surfaces (Figs. 3(a2) and 3(b2)). A cluster of the cactus *Torch* has 18 to 25 spines (Fig. 3(a2)). Their lengths range from 3 to 9 mm. Each spine includes two portions: conical and uniform (Fig. 3(a3)). For ease of manipulation, only the spines with lengths longer than 7 mm are chosen for our tests. The conical portions of the tested spines have the lengths between 4.8 and 6.2 mm. These portions are not ideally straight, which causes an error of 0.5° in measuring conical angles. The conical angles range from 3° to 4°. The uniform portions vary from 2.2 to 2.8 mm in their lengths. They have almost identical cross-sections with the diameters of about 300 μm. As in the cases of the aforementioned three cacti, a *Torch* spine also has microgrooves on its surface (Figs. 3(a4) and 3(a5)). Nevertheless, different from those three cacti, cactus *Torch* does not have any microbarbs on its spine surface.

Each cluster of the cactus *Consolea falcate* contains two to six short or long spines (Fig. 3(b2)). The long spines have lengths in the range of 16 to 22 mm, while the short ones have lengths less than 10 mm. These long spines are adopted in our tests. Each long spine also includes conical

and uniform portions (Fig. 3(b3)). The conical part of a long spine is about 4.7 to 7.5 mm in its length. It also has a conical angle in the range of 3° to 4° with a measurement error of 0.5°. The uniform portion of a long spine has a length varying from 11.3 to 14.5 mm with a diameter of around 330 μm. Similar to cacti *O. microdasys*[1], *Opuntiaengelmannii var. lindheimeri*[12] and *Gymnocalycium baldianum*[13], a *Consolea falcate* spine also has microbarbs and microgrooves on its surface (Figs. 3(b4)-3(b6)). Its top portion is covered by the microbarbs. The length of this portion is about 30% to 50% that of the conical part. The microgrooves extend from the tip portion to the root of the spine. On the other hand, the *Consolea falcate* spine has different microbarbs. In the cases of cacti *O. microdasys*[1] and *Opuntiaengelmannii var. lindheimeri*[12], the microbarbs have a conical shape, and form oblique angles with the spine surfaces. In contrast, the *Consolea falcate* microbarbs lie on the spine surface, and have approximately identical cross-sections. The microbarbs of *Gymnocalycium baldianum*[13] have different shapes. They appear like scales with cavities on the outer surfaces.

The total lengths of spines were measured using a ruler, while the other geometric dimensions were obtained using measuring function of an SEM (model: Hitachi S-3000N) during the process of examining the spines using the SEM.

Measured contact angles

Receding and advancing contact angles were measured using an approach similar to that of ref. 23. Previously, we have used this approach to measure contact angles of a liquid, such as water, on a solid plate.[24] As shown in Fig. S3, a cactus spine was inserted into water. When the spine was stationary inside water, an equilibrium contact angle of water on the spine was observed

through an optical microscope. The pictures of air/water interfaces were subsequently taken using Minisee software of the ScopeTek Company. The contact angles of these interfaces with the spine surface were then determined using MB-Ruler software of the Dance Patterns Company. The advancing and receding angles of the air/water interface on a cactus spine were measured by slightly moving the spine up and down in the solution. In this work, three measurements were taken for each contact angle. It was difficult to identify exact time instant that the interface began to move, which yielded an error of 2° in measuring advancing and receding angles. Table 2 gives the mean values of the contact angles that were measured on representative samples. As observed from this table, untreated *Torch* spines are hydrophilic, while Teflon-coated spines of both cacti are hydrophobic. Meanwhile, untreated spines of *Consolea falcate* have receding and advancing contact angles of 60° and 97°, respectively. Since these angles are less and greater than 90°, respectively, it is difficult to simply say that the untreated spines of *Consolea falcate* is hydrophilic or hydrophobic.

In addition, to avoid cross-contamination, different liquids were tested on different spine samples. At room temperature, water and IPA have surface tensions of 72.8 and 21.7 mN/m, respectively. The wetting degree of a liquid on a solid surface increases with the decrease in surface tension of the liquid. Accordingly, contact angles of water/IPA mixtures should decrease with the increase in the IPA concentration. As observed from Table 2, this is generally true. On the other hand, there may still be some variation, since these mixtures were tested on different spine samples, which were not identical in their surface geometry. For example, in the case of untreated Torch spines, the water/IPA mixture with 75% IPA had smaller receding and advancing contact angles than the one with 100% IPA.

Drop movements in fog tests

At room temperature (24 °C ± 1 °C), two humidifiers (models: EE-5301 and EE-5302, Crane USA Co.) were connected together to generate enough mist to cover a cactus spine (Fig. S4). A plastic pipe is employed to guide this mist flow. A fan (model: Breeze color USB Desktop fan, Arc-tic USA Co.) is used at 800 rounds per minute to increase the mist flow speed to 1.1 m/s, which is measured using a wind speed meter (model: WM-2 Handheld Weather meter, AmbientWeather USA Co.). The humidifiers were turned on for 1 min to ensure that the flow rate was steady. Subsequently, a cactus spine was placed in the mist flow, followed by the recording of the water-collection process through an optical microscope. The flow was perpendicular to the cactus spine.

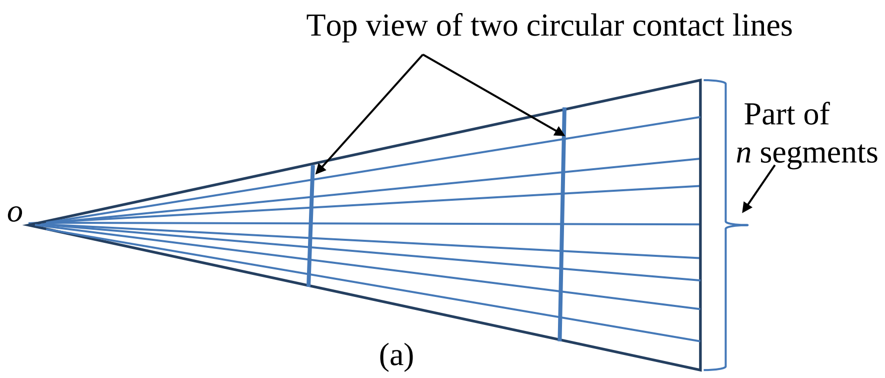

(a)

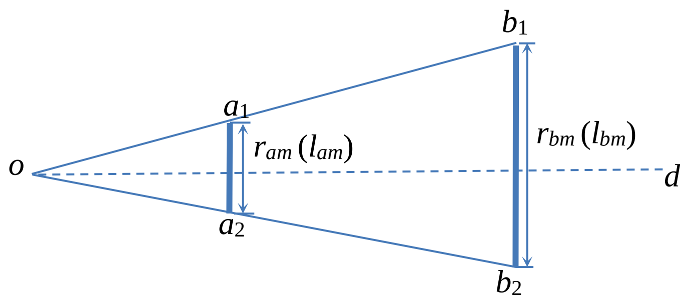

(b)

Figure S1: (a) Divide the whole wire surface into *n* segments for a barrel drop (top view), and (b) consider a segment, which includes the segments a_1a_2 and b_1b_2 of two circular contact lines.

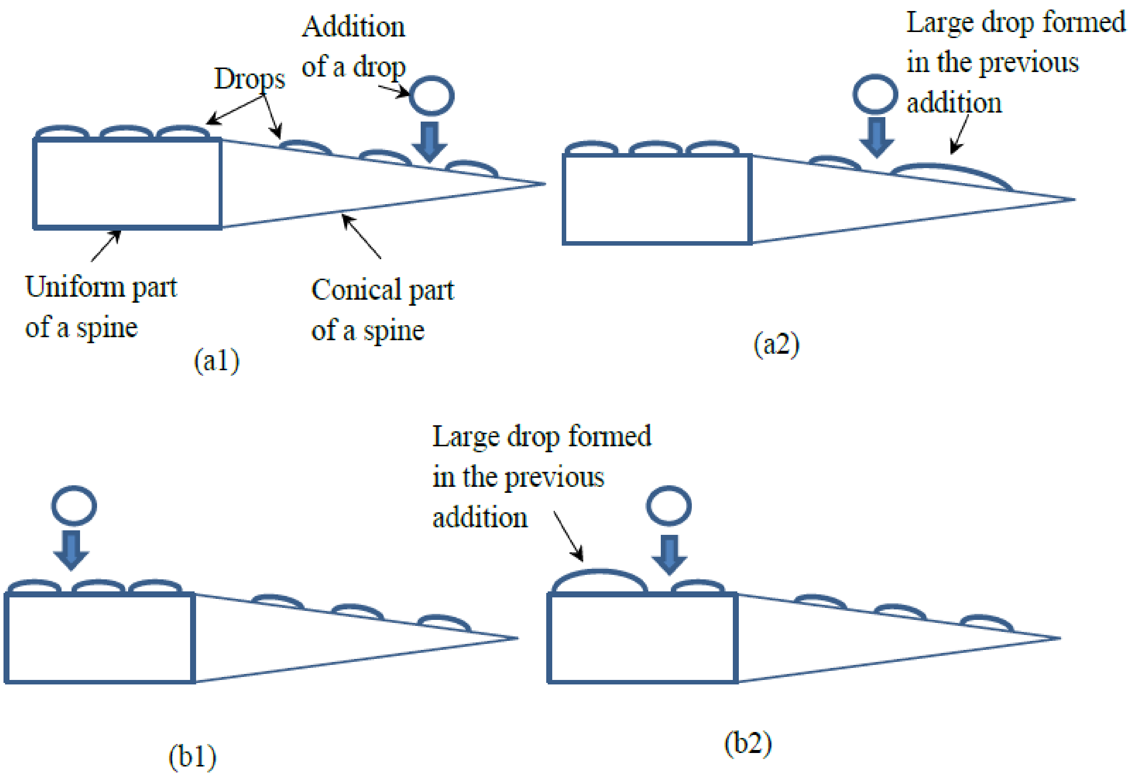

Figure S2: Designs of two tests in the second group of experiments to examine drop movements on a cactus spine (schematics). (a1) and (a2): The first test is to add a water drop between two neighboring drops step-by-step from the tip to the root of a spine. (b1) and (b2): The second test is to do so from the root to the tip of a spine.

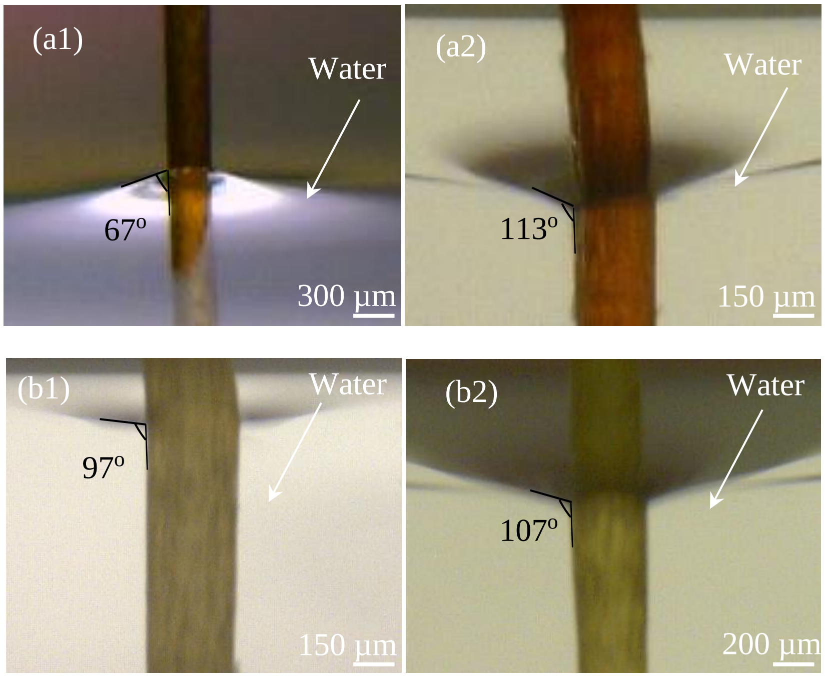

Figure S3: Water on (a1) untreated and (a2) Teflon-coated *Torch* spines, and (b1) untreated and (b2) Teflon-coated *Consolea falcate* spines.

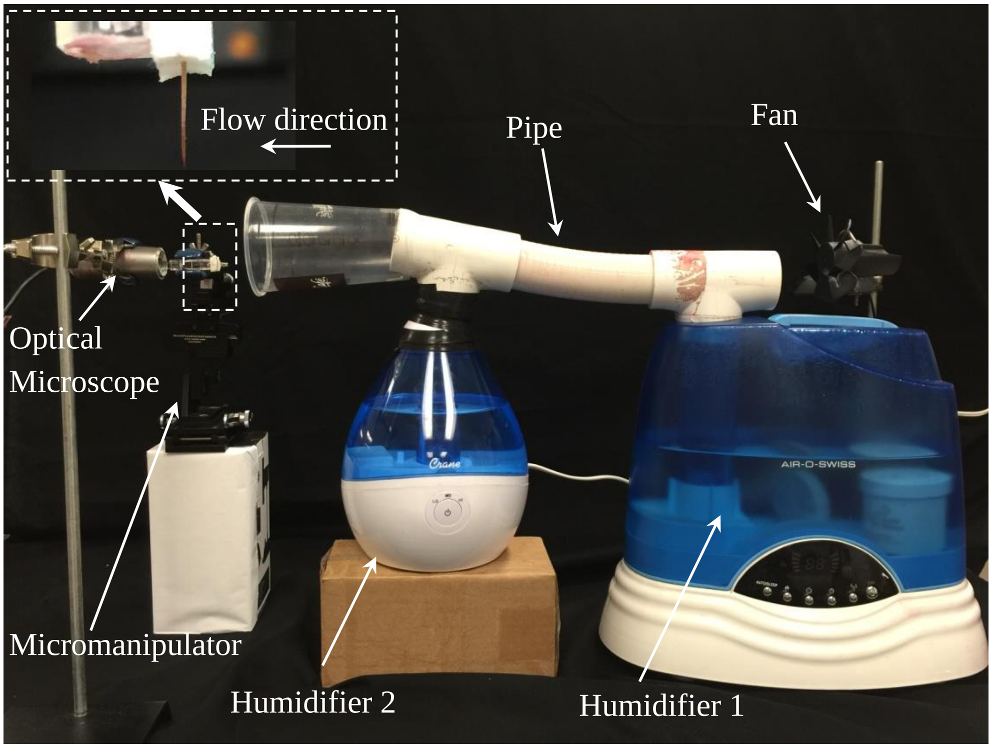

Figure S4: Experimental setup to generate a mist flow over a cactus spine. Insert: top view of the sample location.

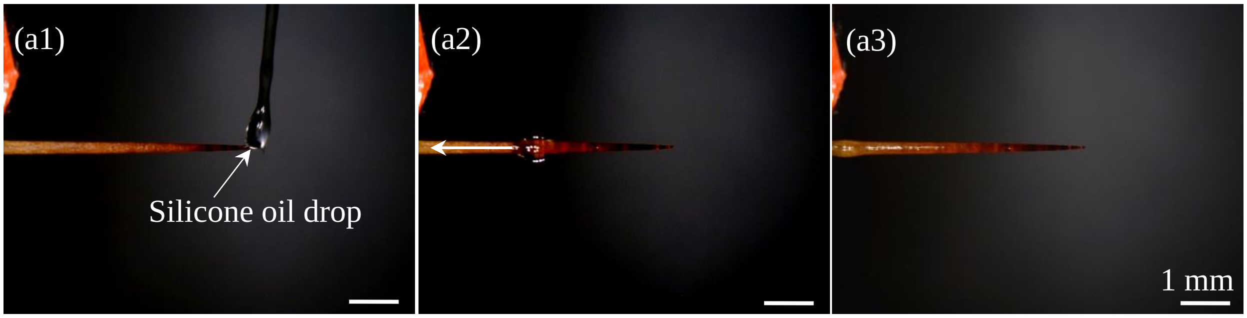

Figure S5: Transport of a silicone oil drop on an untreated *Torch* spine: (a1) the drop was released on the tip of an untreated *Torch* spine, (a2) it self-ran towards the root of the spine, and (a3) it finally stopped at the root. This motion belongs to the first type of movements.

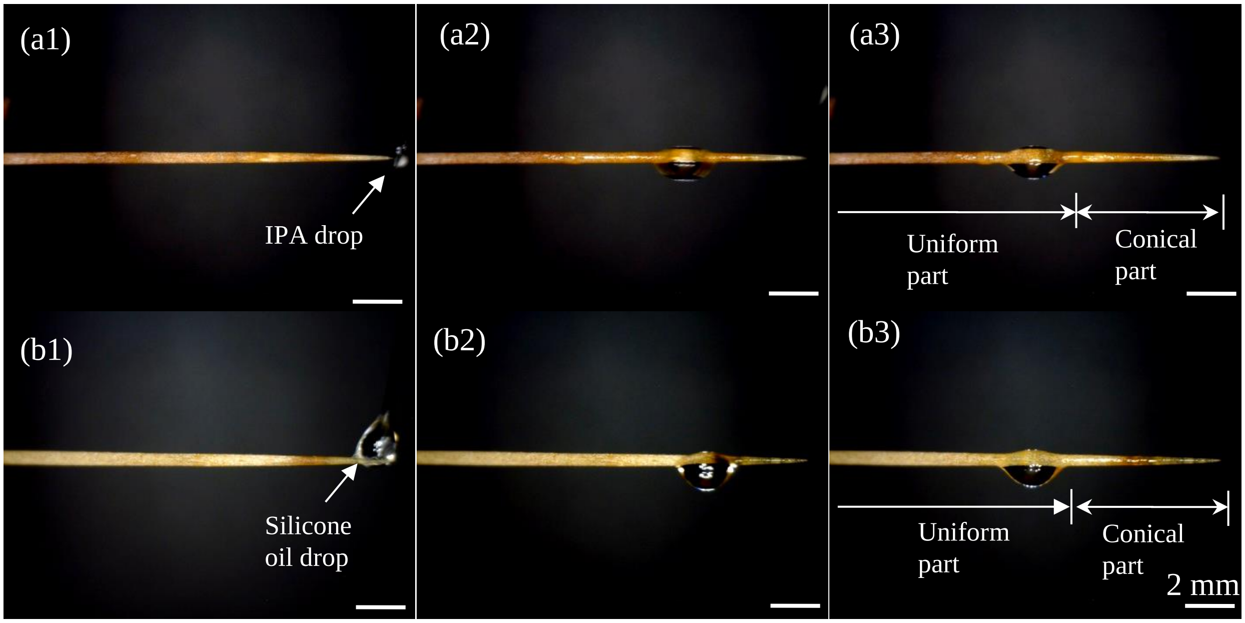

Figure S6: (a1)-(a3) IPA and (b1)-(b3) silicone oil drops transported all the way on the untreated spines of *Consolea falcat* until these drops got to the end of the conical parts. This motion also belongs to the first type of movements.

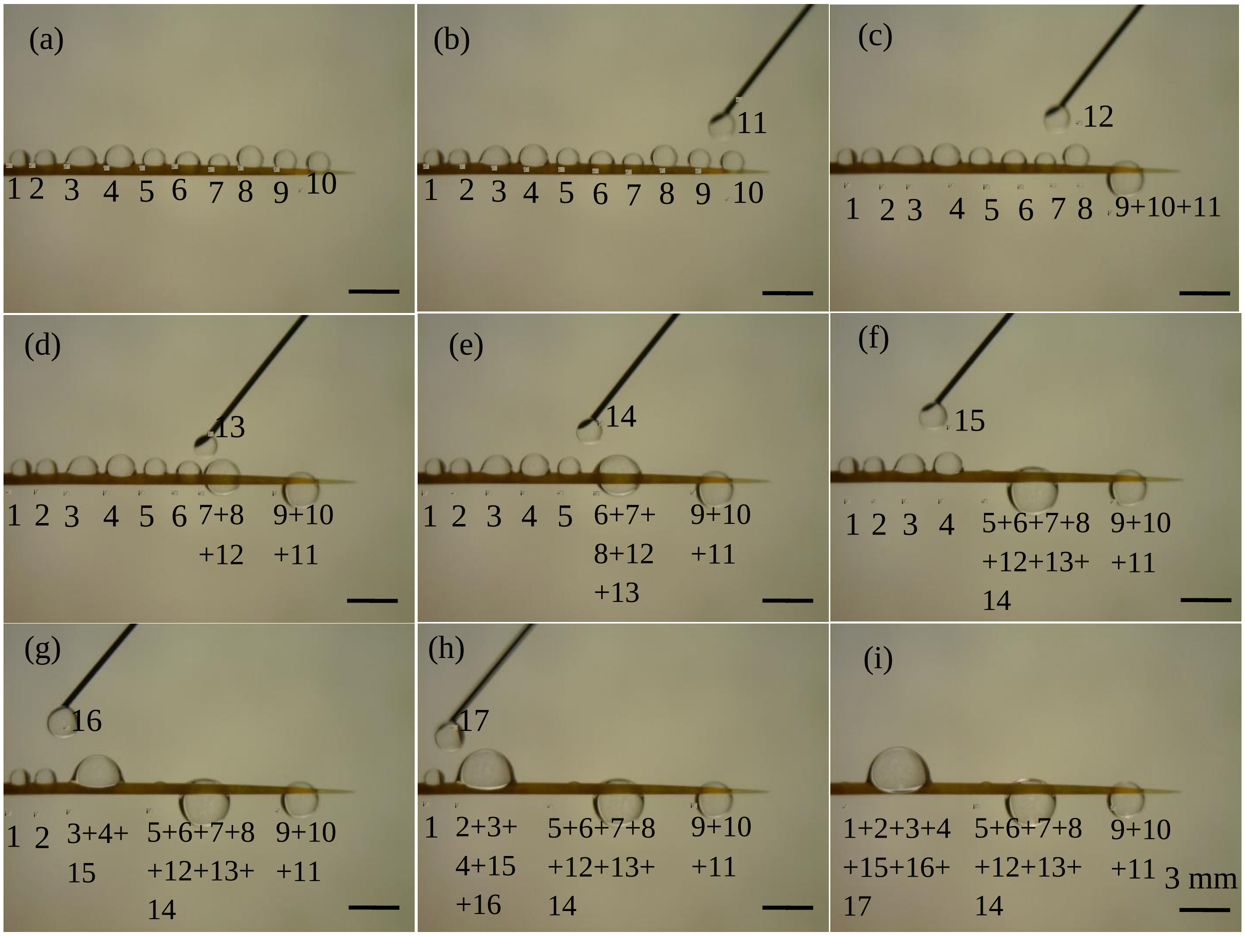

Figure S7: Representative results of the first test of the second group of experiments on an untreated *Consolea falcate* spine. The drops have the same behavior as the one in Fig. 7 for the first test on a Teflon-coated *Torch* spine.

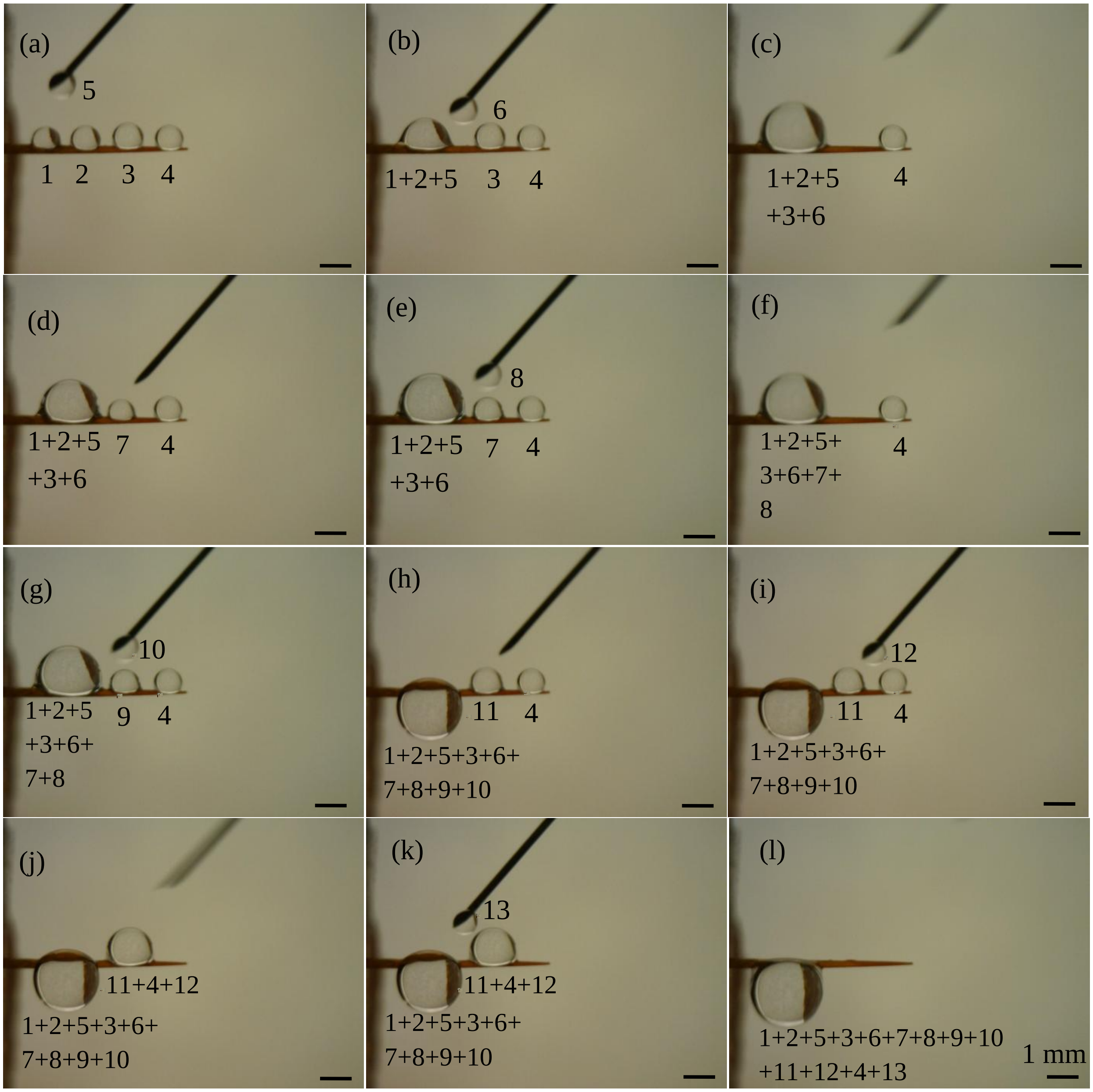

Figure S8: Representive results of the second test of the second group of experiments on an untreated *Torch* spine. The drops have behaviors similar to those in Fig. 6 for the first test on an untreated *Torch* spine. The only difference is that an additional water drop “8” has to be added, for example, between drops “1+2+5+3+6” and “7” in (d) to make them eventually merge together.

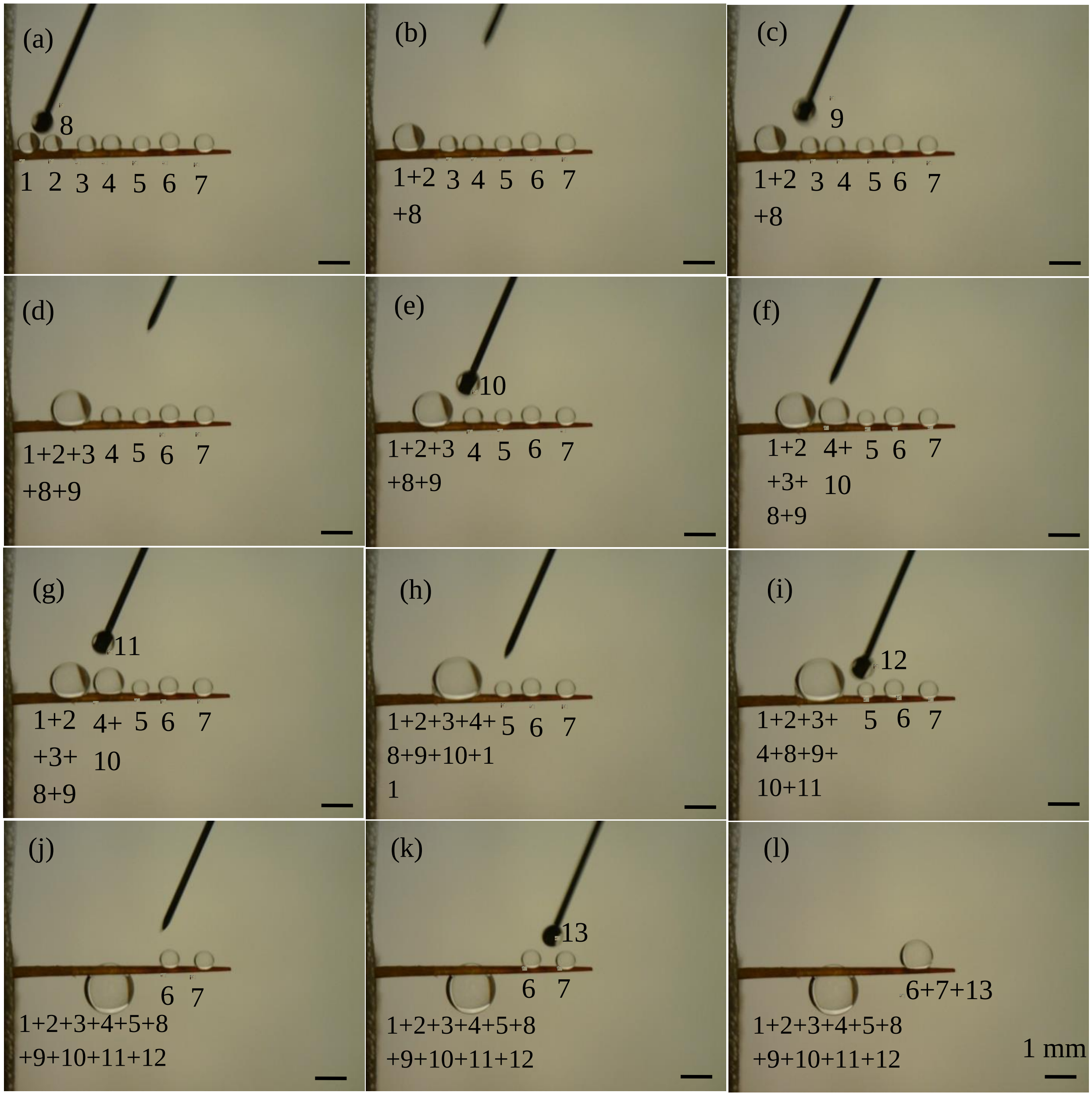

Figure S9: Representative results of the second test of the second group of experiments on a Teflon-coated *Torch* spine. The drops have the same behavior as the one in Fig. 7 for the first test on a Teflon-coated *Torch* spine.

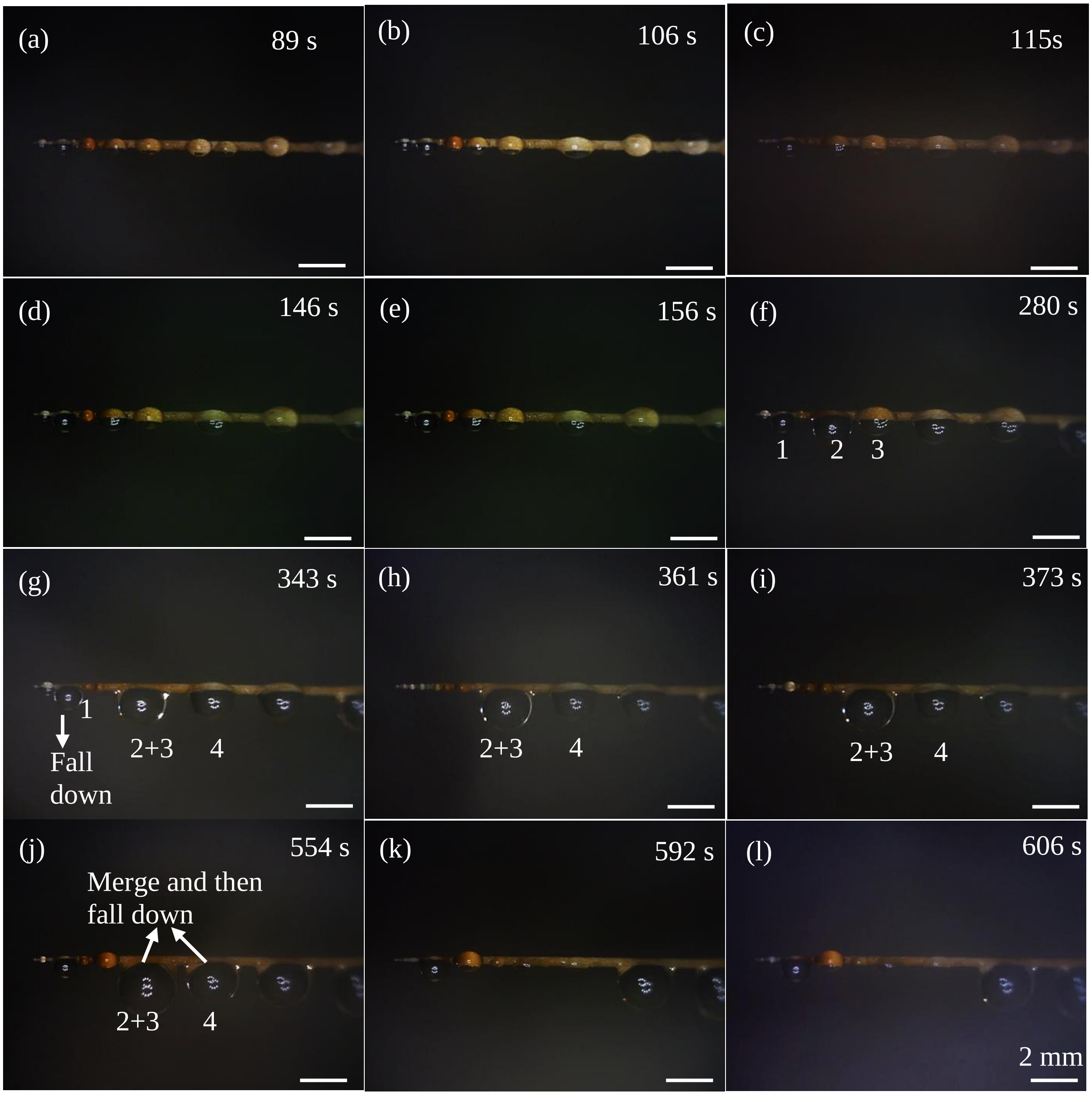

Figure S10: Drop movements on an untreated *Consolea falcate* spine during a fog test, which are similar to those on a Teflon-coated *Torch* spine in Fig. 9. A special point here is that drops are relatively easier to fall off from the spine after the coalescence. This motion belongs to the third type of movements.

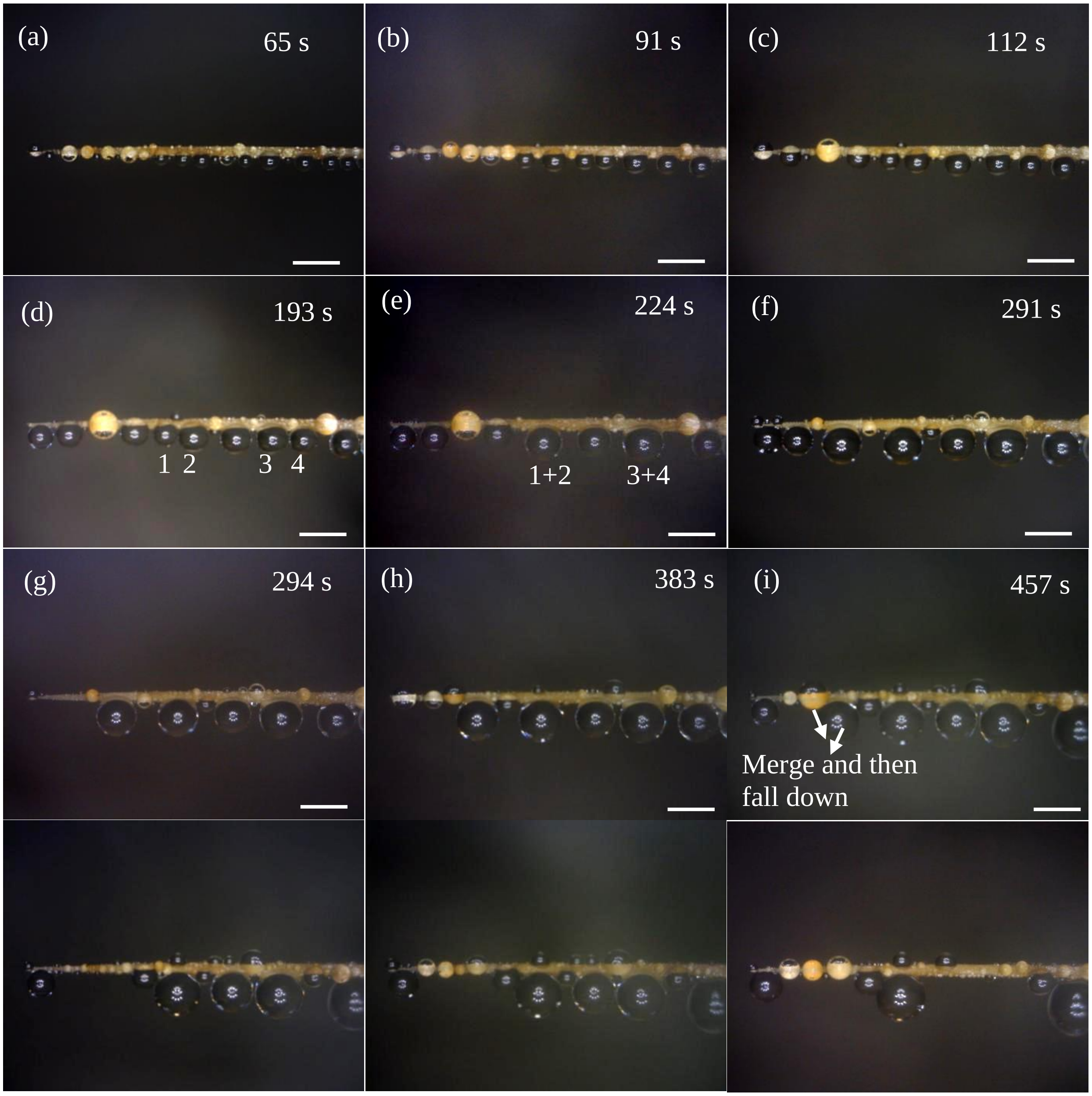

Figure S11: Drop movements on a Teflon-coated *Consolea falcate* spine during a fog test, which are similar to those on a Teflon-coated *Torch* spine in Fig. 9. A special point here is also that drops are relatively easier to fall off from the spine after the coalescence. This motion also belongs to the third type of movements.

Video 1: This video corresponds to Fig. 6 in the main article, and it shows behavior of multiple water drops on an untreated *Torch* spine. The corresponding motion belongs to the second type of movements.

Video 2: This video is related to Fig. 7 in the main article, and it demonstrates behavior of multiple water drops on a Teflon-coated *Torch* spine. The corresponding motion belongs to the third type of movements.
